# An Early Neoplasia Index (ENI10), Based on Molecular Identity of CD10 Cells and Associated Stemness Biomarkers, is a Predictor of Patient Outcome in Many Cancers

**DOI:** 10.1158/2767-9764.CRC-23-0196

**Published:** 2023-09-29

**Authors:** Boris Guyot, Flora Clément, Youenn Drouet, Xenia Schmidt, Sylvain Lefort, Emmanuel Delay, Isabelle Treilleux, Jean-Philippe Foy, Sandrine Jeanpierre, Emilie Thomas, Janice Kielbassa, Laurie Tonon, Helen He Zhu, Pierre Saintigny, Wei-Qiang Gao, Arnaud de la Fouchardiere, Franck Tirode, Alain Viari, Jean-Yves Blay, Véronique Maguer-Satta

**Affiliations:** 1 CNRS UMR5286, Centre de Recherche en Cancérologie de Lyon, Lyon, France.; 2 Inserm U1052, Centre de Recherche en Cancérologie de Lyon, Lyon, France.; 3 Department of Cancer Initiation and Tumor cell Identity, Centre de Recherche en Cancérologie de Lyon, Lyon, France.; 4 Universite Claude Bernard Lyon 1, CRCL, Lyon, France.; 5 Centre Léon Bérard, Lyon, France.; 6 Department of Tumor Escape Resistance and Immunity, CRCL, Lyon, France.; 7 Bioinformatics Platform, Synergie Lyon Cancer Foundation, Lyon, France.; 8 State Key Laboratory of Oncogenes and Related Genes, Renji-Med-X Stem Cell Research Center, Shanghai Cancer Institute and Department of Urology, Ren Ji Hospital, School of Medicine and School of Biomedical Engineering, Shanghai Jiao Tong University, Shanghai, P.R. China.; 9 School of Biomedical Engineering and Med-X Research Institute, Shanghai Jiao Tong University, Shanghai, P.R. China.

## Abstract

An accurate estimate of patient survival at diagnosis is critical to plan efficient therapeutic options. A simple and multiapplication tool is needed to move forward the precision medicine era. Taking advantage of the broad and high CD10 expression in stem and cancers cells, we evaluated the molecular identity of aggressive cancer cells. We used epithelial primary cells and developed a breast cancer stem cell–based progressive model. The superiority of the early-transformed isolated molecular index was evaluated by large-scale analysis in solid cancers. BMP2-driven cell transformation increases CD10 expression which preserves stemness properties. Our model identified a unique set of 159 genes enriched in G_2_–M cell-cycle phases and spindle assembly complex. Using samples predisposed to transformation, we confirmed the value of an early neoplasia index associated to CD10 (ENI10) to discriminate premalignant status of a human tissue. Using a stratified Cox model, a large-scale analysis (>10,000 samples, The Cancer Genome Atlas Pan-Cancer) validated a strong risk gradient (HRs reaching HR = 5.15; 95% confidence interval: 4.00–6.64) for high ENI10 levels. Through different databases, Cox regression model analyses highlighted an association between ENI10 and poor progression-free intervals for more than 50% of cancer subtypes tested, and the potential of ENI10 to predict drug efficacy. The ENI10 index constitutes a robust tool to detect pretransformed tissues and identify high-risk patients at diagnosis. Owing to its biological link with refractory cancer stem cells, the ENI10 index constitutes a unique way of identifying effective treatments to improve clinical care.

**Significance:**

We identified a molecular signature called ENI10 which, owing to its biological link with stem cell properties, predicts patient outcome and drugs efficiency in breast and several other cancers. ENI10 should allow early and optimized clinical management of a broad number of cancers, regardless of the stage of tumor progression.

## Introduction

Originally identified in leukemia as a tumor-specific antigen, CD10 (encoded by the *MME* gene) is associated with multiple cellular functions in normal and pathologic contexts (1). This cell-surface zinc-dependent endopeptidase is expressed on normal stem cells (SC) and involved in their regulation through the cleavage of peptides from the microenvironment (2–4). CD10 expression characterizes sphere-forming cells in several human primary tissues and contributes to maintaining immature properties of the mammary gland by controlling SC fate and preventing differentiation (5). CD10 expression is increasingly associated with cancer stem cells (CSC; refs. 6–11). At the clinical level, CD10 may be a marker of both good and poor prognosis, likely related to the stage of the cancer, the cell type expressing it, the tissue of origin, the associated factor explored and clinical treatment (1, 6, 8, 12–15). The functional link between CD10 at the (stem) cell membrane and the bone morphogenetic proteins (BMP) differentiation signal has been identified in different processes (16–18). We and others revealed the implication of BMP signaling in the transformation of hematopoietic (19, 20) or epithelial (21–23) SCs. This small population of cells that self-renew and differentiate into other cancer cell types is associated with tumor heterogeneity, progression, metastatic dissemination, and resistance to treatment (24). Identifying such BMP-responding SCs (i.e., through CD10 expression) and deciphering their transformation mechanism, may improve their targeting and eradication. However, the detection of CSC at an early stage to predict tumor aggressiveness and adapt therapeutic strategies remains a challenge as CSC are difficult to identify and distinguish from their normal counterparts (25, 26).

Here, we evaluated the importance of CD10-expressing cells in early stages of SC transformation driven by the bone morphogenetic protein 2 (BMP2). We revealed that, while CD10 expression increases during BMP2-driven mammary epithelial transformation and is characteristic of cell populations with stemness properties, it does not impact the transformed phenotype. After transformation cells retained their SC properties and the molecular identity related to the CD10 control of key elements of the asymmetric division machinery. Consequently, we derived from our results a unique molecular tool (ENI10 score) applicable to a broad range of tumors for the early detection of transformation and patient follow-up, to predict survival and potentially support therapeutic choices.

## Materials and Methods

### Animal Experimentation

Animal experiments were authorized by the ethics committee for animal experimentation of the Rhône-Alpes region (CECCAPP), France. Following long-term treatment with BMP2 and IL6, 2 or 5 million MCF10A, MC26 or M1B26 cells were mixed with 50% growth factor–reduced Matrigel (BD Biosciences) and injected subcutaneously close to the fourth inguinal mammary gland of 6–7 weeks old athymic nude mice (RRID:IMSR_JCL:MID-0001, Harlan). Ten mice were injected per group. A 10 mg/mL β-estradiol solution was applied to the neck region of the animals twice a week. Tumor formation was monitored by measuring the size of the tumor. Mice were sacrificed after 6 weeks, and tumors were fixed, paraffin-embedded, sectioned, and subjected to hematoxylin and eosin (H&E) staining.

### Human Primary Tissue

The obtention of human tissue samples was approved by the ethics board of the Léon Bérard Cancer Center in accordance with the Declaration of Helsinki guidelines and patients gave written informed consent. Normal and BRCA-mutated human mammary glands were obtained from patients undergoing reduction mammoplasty or prophylactic mastectomy, respectively. Mammary epithelial cells from healthy or BRCA carriers (three BRCA1 and three BRCA2) were prepared by BB-0033-00050, CRB Centre Léon Bérard, Lyon France as described in ref. 5.

### Cell Isolation, Culture, and Breast Cancer Transformation Model

Primary cells were obtained from healthy human adult undergoing breast reduction mammoplasty, BRCA mutations carriers undergoing prophyalactic mastectomy or breast tumors after surgical removal (informed consent was obtained from the patients) as described previously (5, 21). MCF10A cells (RRID:CVCL_0598) were purchased from the ATCC in 2008 (batch 7635052) without additional authentication and cultured according to the manufacturer's recommendations in phenol red–free DMEM/F-12 nutrient mix supplemented with 5% horse serum (Life), 10 µg/mL insulin, 0.5 µg/mL hydrocortisone, 100 ng/mL cholera toxin and 20 ng/mL EGF (all supplied by Sigma), 1% penicillin/streptomycin (Life Technologies). Exposure of MCF10A cells to BMP2 and IL6 (both at 10 ng/mL) led to the generation of the MC26 cell line that mimics luminal breast tumors (21). Because we showed that BMP2-mediated transformation was dependent on bone morphogenetic proteins receptor 1B (BMPR1B) expression, we also used sorted BMPR1B^+^ MCF10A cells, in that case transformation was observed after only a few weeks of BMP2 and IL6 treatment. Three soft-agar clones from these BMP2/IL6-treated BMPR1B^+^ MCF10A cells were selected and expanded in the presence of BMP2/IL6, giving rise to the M1B26 cell line. Absence of *Mycoplasma* was routinely tested by PCR in all cell lines.

### Functional Assay in Cell Lines

For mammosphere assays, single cells were seeded onto 96-well ultra-low attachment plates (BD Corning) at limiting dilutions (100 cells/well) for 7 days using the described sphere assay protocol (21). Resulting spheres were counted. For the epithelial colony-forming cell (E-CFC) assay, cells were seeded in MCF10A 2% serum medium at a limiting dilution (250 cells/well of a 12-well plate) on an irradiated fibroblast layer for 7 days, and resulting colonies were counted and classified using size and shape criteria. For three-dimensional (3D) terminal duct lobular units (TDLU) assays, 500 cells were seeded in growth factor–reduced Matrigel (BD Corning) and assays were carried out in complete medium (22). Analysis of 3D structures and all other assays were performed using Axiovert 25 microscope (RRID:SCR_002677), and images were analyzed with the AxioVision 4.6 software (AxioVision Imaging System). Structures were then washed with PBS 1X, fixed using formaldehyde 1% for 2 hours, and sent to the AniPATH facility (Lyon) for inclusion, section and H&E staining.

### Soft-Agar Colony Formation

To evaluate the transformation of cells, soft-agar colony formation assays were performed as follows: the bottom agar layer was prepared from 1.8% agar (Promega) diluted in an equal volume of 2X culture medium to a final concentration of 0.9%, added to cell culture plates and incubated at room temperature for 30 minutes. The top agar layer was prepared accordingly at a final density of 0.45%. Cells were mixed into the liquid top agar and added on top of the bottom agar at a final concentration of 10,000 cells/mL. Colonies were quantified and measured after 15 to 21 days of culture at 5% CO_2_ and 37°C.

### Retroviral Production and Infection

The CMV-BMP2-mPGK-hygromycin lentiviral vector construct and its corresponding control were a gift from Dr R. Iggo, University of Bordeaux, France. The pLenti X2 Puro empty control vector (RRID:Addgene_20957) and the pLenti X2 puro DEST (RRID:Addgene_17296) used to clone the pX2-shBMPR1B vector were purchased from Addgene. Lentiviruses were produced by calcium phosphate cotransfection of lentiviral constructs with a VSV-G envelope construct (pMD2.G, RRID:Addgene_12259) and gagpol packaging construct (PCMV-dR8.74) into HEK293T cells (RRID:CVCL_HA71) according to standard techniques. Medium was replaced 6 hours after transfection. Lentiviral particles were collected 48 hours after transfection. Lentiviral titers were determined for each viral batch by serial dilution infections of MCF10A cells and subsequent puromycin or hygromycin B (both from Sigma-Aldrich) treatment. MCF10A cells were seeded one day prior to infection and cells were infected overnight at a multiplicity of infection of 5–10. Forty-eight hours after infection, transduced cells were selected by puromycin or hygromycin B treatment for 96 hours to 2 weeks.

### qRT-PCR

RNA was extracted using the RNeasy Plus Mini Kits (Qiagen) containing a gDNA eliminator column or TriReagent (Sigma-Aldrich) and chloroform extraction using Phase Lock Gel columns (5Prime). RNA concentration was measured on a Nanodrop ND-1000 spectrophotometer (RRID:SCR_016517). Reverse transcription was conducted using Superscript II (Invitrogen) according to the manufacturer's instructions. cDNA was stored at −80°C. Quantitative PCR (qPCR) was performed using sequence-specific primers on a LightCycler 480 II system (RRID:SCR_020502, Roche Applied Science) with SyBR Green I technology (QuantiFAST SyBR kit from Qiagen) and LightCycler 480 Multiwell Plate 96 (Roche Applied Science). *CPB* and *ACTB1* were selected by geNORM (RRID:SCR_006763) analysis as reference genes.

### Flow Cytometry and Cell Sorting

Cells were resuspended in PBS and incubated for 30 minutes to 1 hour with 8 µL of PE-conjugated anti-CD10 (HI10a clone, mouse IgG1κ, RRID:AB_396586, BD Biosciences) per 10^6^ cells. After centrifugation, cells were resuspended in HBSS, 2% FBS for flow cytometry cell sorting at a concentration of 5–10 × 10^6^ cells/mL. Cell sorting was performed using a FACS Aria cell sorter (RRID:SCR_019595, BD Biosciences) at low pressure (psi: 20) with 488 and 633 nm lasers. For phenotypic analysis, cells were suspended in PBS 1X and incubated for 30 minutes to 1 hour with 1 µL PE-conjugated anti-CD10 antibody or PE-conjugated isotype (MOPC-21 clone; mouse IgG1κ, RRID:AB_394195) from BD Biosciences. Flow cytometry was performed using a FACSCalibur cell analyzer (RRID:SCR_000401, BD Biosciences) and analyzed using the FlowJo software (RRID:SCR_008520).

### Transcriptomic Analysis

Microarray analysis was performed by the platform ProfileXpert (SFR Santé Lyon‐Est UCBL-UMS 3453 CNRS – US7 INSERM) using a high-density oligonucleotide array (GeneChip Human Genome U133 plus 2.0 array, Affymetrix). Total RNA (50 ng) from healthy human adult breast reduction mammoplasty cells, or BRCA carriers were amplified and biotin-labeled using GeneChip 3′ IVT PLUS kit. Before amplification, spikes of synthetic mRNA at different concentrations were added to all samples; these positive controls were used to ascertain the quality of the process. Biotinylated antisense cRNA for microarray hybridization was prepared. After final purification using magnetic beads, cRNA quantification was performed on a Nanodrop and quality checked with an Agilent 2100 Bioanalyzer (Agilent Technologies, Inc). Hybridization was performed following the Affymetrix protocol. Briefly, 10 µg of labeled cRNA was fragmented and denaturated in hybridization buffer, then hybridized on chip for 16 hours at 45°C with constant mixing by rotation at 60 rpm in a Genechip hybridization oven 640 (RRID:SCR_019346, Affymetrix). After hybridization, arrays were washed and stained with streptavidin-phycoerythrin (GeneChip Hybridization Wash and Stain Kit) in a fluidic 450 (RRID:SCR_018034, Affymetrix) according to the manufacturer's instruction. The arrays were read with a confocal laser (Genechip scanner 3000, RRID:SCR_016522, Affymetrix). CEL files were then generated using the Affymetrix GeneChip Command Console (AGCC) software 3.0. Identification of the genes composing the CD10 signature was conducted using the GenePattern modules (27). Briefly, CEL files were converted to RES files using the “ExpressionFileCreator module”, log_2_ transformed using the “PreprocessDataset” module and different probe set values for a gene were converted to a single value by the “CollapseDataset” module using the “maximum” collapse mode. Differentially expressed genes between CD10^−^ and CD10-positive (CD10^+^) MCF10A-CT cells were then identified using the “ComparativeMarkerSelection” module.

For RNA sequencing (RNA-seq) analysis, total RNA was extracted using the RNeasy Mini Kit Plus (Qiagen). Poly-A RNA libraries were prepared for sequencing using standard Illumina reagent and procedures and paired-end sequenced on an Illumina NovaSeq6000 apparatus (RRID:SCR_016387). Raw sequencing reads were aligned on the human genome (GRCh38) with STAR (v2.7.3a, RRID:SCR_004463), with the annotation of known genes from gencode v33. Gene expression was quantified using Salmon (1.1.0) and the annotation of protein coding genes from gencode v33.

### Bioinformatics Analysis

Data analysis was performed using the Array Studio software (Omicsoft Corporation) and the Bioconductor (RRID:SCR_006442) packages in the R language (http://www.bioconductor.org; ref. 28). Raw data from microarrays were processed using quantile normalization and the robust multiarray average (RMA) algorithm and were log_2_ transformed.

Gene set enrichment analysis (GSEA) was performed using the “preranked” tool (29). The single-sample GSEA (ssGSEA) function of the GSVA package from Bioconductor or the ssGSEA 2.0 package (30) was used to compute separate scores for each sample of a given dataset using the ENI10 signature or other gene sets derived or not from ENI10 that are described in the Results section.

Analysis of the melanoma cohort was done thanks to the RNA-seq data acquired during routine molecular diagnosis performed at the Centre Léon Bérard Cancer Center. RNA-seq data from this cohort are available on simple request. Expression values were extracted using Kallisto version 0.42.5 tool with GENCODE release 23-genome annotation based on GRCh38 genome reference. Kallisto transcript per million (TPM) expression values were transformed in log_2_(TPM+2) and all samples were normalized together using the quantile method from the R LIMMA package within R (version 3.1.2) environment.

The results shown here are in whole or part based upon data generated by The Cancer Genome Atlas (TCGA) Research Network: https://www.cancer.gov/tcga (RRID:SCR_003193). TCGA RNA data were obtained from the GDC data portal available at https://portal.gdc.cancer.gov/. Curated clinical data were obtained from Supplementary Table S1 of TCGA-CDR article (31). Following the author's recommendations, we used progression-free interval (PFI) as the outcome endpoint for survival analysis excepted for acute myeloid leukemia (LAML) cancers for which overall survival (OS) was used. PAM50 breast cancer subtypes for TCGA-BRCA samples were obtained from additional file 2 of the following article (32), where the normal-like samples were removed because this subtype is likely to be an artifact caused by normal cells contamination of the tumor (33).

### Statistical Analysis

Data from the different MCF10A cell–derived models were compared using the paired Student *t* test, when data were normally distributed, or the Wilcoxon signed-rank test when data were not normally distributed. Unpaired Student *t* test or Mann–Whitney test were performed to compare continuous data between two groups and one-way ANOVA or Kruskal–Wallis test if more than two groups. Pearson χ^2^ test or Fisher exact test were used to analyze qualitative data. OS as well as progression-free survival (PFS) curves were estimated using the Kaplan–Meier method and compared with the log‐rank test between groups of patients defined by the median of the signature enrichment scores (low vs. high score). For TCGA data analysis, the effect of the ENI10 score on survival outcome was estimated, for each cancer separately, by HRs corresponding to one SD of the ENI10 score taken as a continuous variable in the Cox model. To obtain an “overall Pan-Cancer” estimate of the effect of the ENI10 score, unadjusted and multivariable Cox models were fitted with a strata term on cancer type (i.e., each tumor type had a specific baseline hazard function) so that variations in survival between the different cancers were taken into account and treated as a “nuisance parameter”. For this Pan-Cancer analysis, the ENI10 score was discretized into deciles, to finely investigate a putative dose–response relationship of the effect of the ENI10 score on survival outcome. To compare the ENI10 score levels in tumor and normal paired samples, the Wilcoxon signed-rank test was used. All statistical tests were two sided, and *P* values <0.05 were considered to be statistically significant. The statistical analysis was performed using GraphPad Prism version 6.00 (RRID:SCR_002798) and Bioconductor packages in the R language.

### Data Availability

Transcriptomic data were deposited on the Gene Expression Omnibus repository under the accession numbers GSE123053 (for the microarray data on CD10 sorted cell lines), GSE186734 (for the RNA-seq data on unsorted cell lines), GSE186733 (for the RNA-seq data on healthy or BRCA-mutated primary human epithelial cells), and GSE186735 (for the RNA-seq data on shCD10-expressing MCF10A cells).

## Results

### CD10 Expression and BMP2-driven Mammary SC Transformation

**FIGURE 1:**
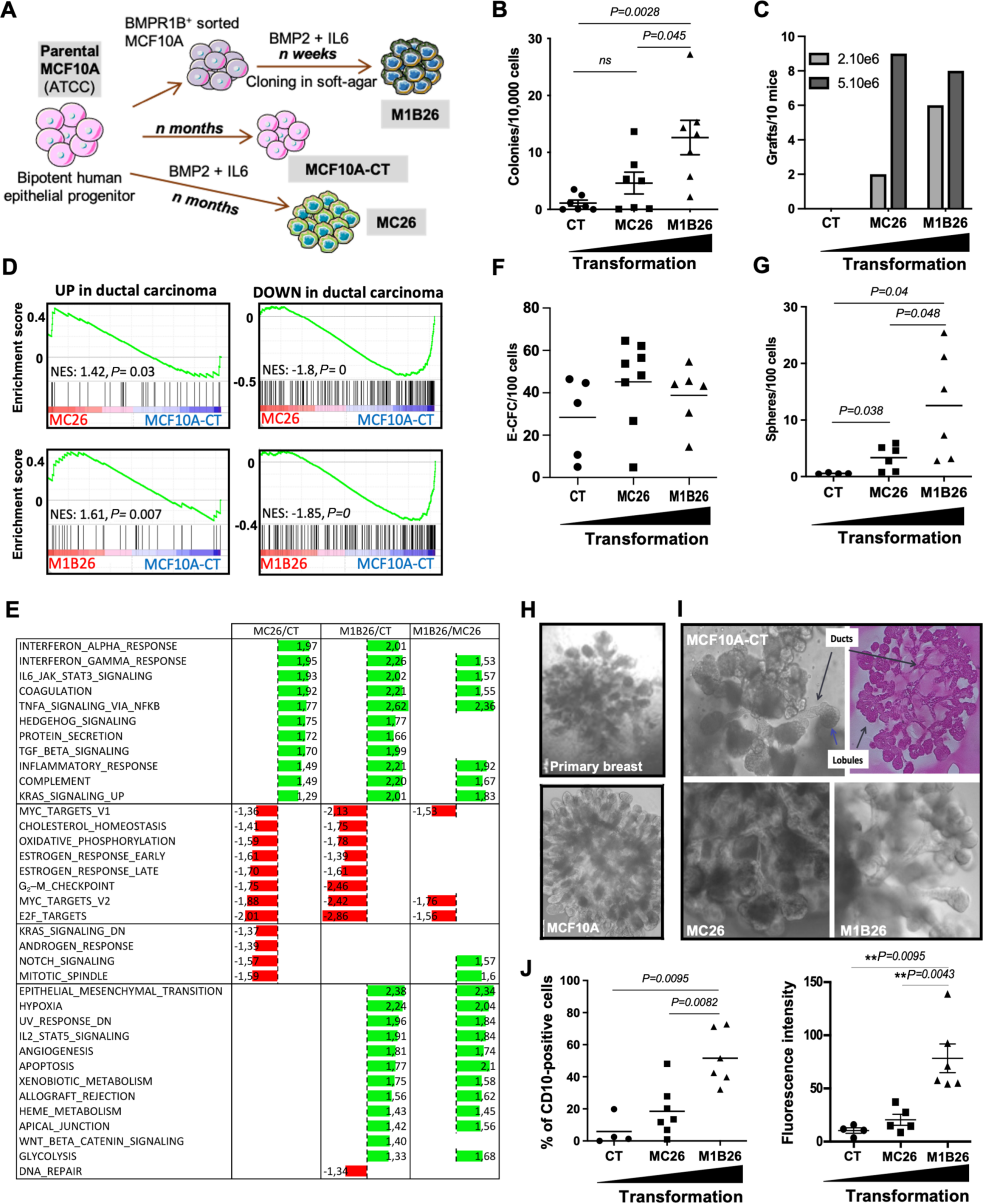
BMP2 induces early transformation of a model of breast SCs and increases CD10 expression. **A,** Schematic representation of the experimental protocol used to obtain the MCF10-CT, MC26, and M1B26 cell lines from the MCF10A cells. **B,** Quantification of soft-agar colony formation [error bars represent the SD (*n =* 7), significance measured using the Mann–Whitney test is indicated on the graph by the *P* values. *ns for P >* 0.05]. **C,** Xenografts of the indicated number cells of MCF10A-derived models injected into nude mice presented as the number of successful grafts after 4 weeks/mouse (*n* = 10). **D,** GSEA of transcriptomic data comparing MC26 cells (top row) or M1B26 cells (bottom row) with MCF10A-CT cells. Data represent enrichment plots analyzed using public gene sets (41) of upregulated (left) or downregulated (right) genes in human primary ductal carcinoma compared with healthy tissues. **E,** GSEA of transcriptomic data comparing MC26 (first column) or M1B26 (second column) with CT cells and M1B26 with MC26 cells (third column). The “hallmarks” gene sets from the MSigDB were used and the NES (normalized enrichment score) with *P* values inferior to 0.05 are shown. **F,** E-CFC Progenitor content from MCF10A-CT cells (*n* = 5), MC26 cells (*n* = 8) or M1B26 cells (*n* = 6) quantified after 6 days by scoring colonies numbers and presented/10,000 cells. **G,** Number of spheres per 100 seeded cells after 1 week from MCF10A-CT cells (*n = 4*), MC26 cells (*n = 6*), and M1B26 cells (*n = 6*). **H,** TDLU, 3D structures from primary human breast cells (top) and MCF10A cell line (bottom). **I,** Images at day 21 of 3D structures in the TDLU assay from MCF10A-CT, MC26, or M1B26 cells. A representative TDLU section from MCF10A-CT, stained with H&E, is shown on the top right. **J,** Flow cytometry analysis of CD10 expression on MCF10A-CT (*n = 4*), MC26 (*n = 7*), and M1B26 (*n = 6*) presented as the percentage of positive cells (left) and mean fluorescence intensity (right).

To evaluate the early association between CD10 and the first steps of breast cancer development, we developed new human models of breast cancer. We used MCF10A cells [nonmalignant fibrocystic mammary cells, *p16/CDKN2A* deleted, *MYC* amplified (34)] that display immature properties in 3D cultures (35, 36) and like primary human mammary SC reconstruct a duct and lobule 3D structure in TDLU assay (37, 38). Indeed, to avoid any immediate, nonphysiologic, massive and sharp alteration, we chose not to overexpress master oncogenes but used a more physiologic protocol based on a prolonged chronic exposure to soluble factors known to be overproduced in breast cancer and to promote tumorigenesis (21, 39, 40). Hence, based on our previous description that BMP2-transforming effect were mediated by the BMPR1B (21), unsorted or BMPRIB^+^-sorted MCF10A cells were transformed by long-term exposure to BMP2 and IL6 to generate the MC26 or M1B26 cell line, respectively (Fig. 1A). Consistently with our previous finding, BMP2-mediated transformation was much faster on cells sorted for high BMPR1B expression (Fig. 1A). The parental and transformed cells showed similar doubling time albeit M1B26 proliferates slightly slower than MCF10A-CT and MC26 cells (Supplementary Fig. S1A). The relative levels of transformation of the different MCF10A-derived models were then assessed using soft-agar colony formation assays (Fig. 1B) and engraftment assays in immunocompromised mice (Fig. 1C). Results indicated that both MC26 and M1B26 cells have an increased ability to form anchorage-independent clones and were able to engraft in mice, compared with untreated control MCF10A cells (CT). In both assays, M1B26 cells displayed a higher level of transformation than MC26 (Fig. 1B and C), suggesting that they constitute novel models of progressive transformation to study early steps of tumorigenesis. A transcriptomic analysis of these cell lines revealed that both MC26 and M1B26 cells present a molecular expression profile highly similar to primary breast cancer cells for upregulated and downregulated genes while MCF10A-CT cells displayed a profile close to normal tissue compared with breast ductal carcinoma or normal breast tissue (ref. 41; Fig. 1D). We then applied a GSEA to the genes differentially expressed between MC26, M1B26, and parental CT cells using hallmark gene sets from the Molecular Signature DataBase (MSigDB; ref. 42). Genes involved in the response to interferon alpha and gamma, in TNFα signaling and genes activated following KRAS signaling were upregulated in MC26 and M1B26 cells compared with CT cells. In addition, genes involved in oxidative phosphorylation were downregulated in the transformed cell lines (Fig. 1E). A complete Gene Ontology (GO) enrichment analysis comparing MC26 and M1B26 cells with MCF10A-CT cells is shown in the Supplementary Table S1. As MCF10A cells display immature properties similar to primary human mammary cells (such as TDLU, sphere and epithelial colony-forming ability as well as molecular immature markers), we assessed the ability of MC26 and M1B26 cells to generate spheres, TDLU and the presence of E-CFCs (22, 37). We observed no differences in the frequency of E-CFC between MCF10A-CT, MC26, and M1B26 (Fig. 1F) and an increase in mammosphere frequency with transformation (Fig. 1G). As for to parental MCF10A cells and primary breast epithelial cells, MC26 and M1B26 models produced TDLU, further demonstrating that these transformed cells retained their immature properties (Fig. 1H and I). In accordance with the expression of CD10 on mammary SCs (5), flow cytometry analysis revealed a higher proportion of membrane CD10^+^ cells in M1B26 (51.6%) and MC26 (18.5%) models compared with CT (5.9%) cells (Fig. 1J, left). Moreover, higher mean fluorescence intensity indicated that CD10^+^ transformed cells also displayed more CD10 molecules per cell than their nontransformed counterparts (Fig. 1J, right).

Collectively, these data indicate that MC26 and M1B26 cells constitute novel models of early steps of progressive transformation associated with an increased CD10 expression.

#### The ENI10 Molecular Signature, Related to CD10^+^ Mammary SCs, Identifies Patients with High-risk Breast Cancer

**FIGURE 2:**
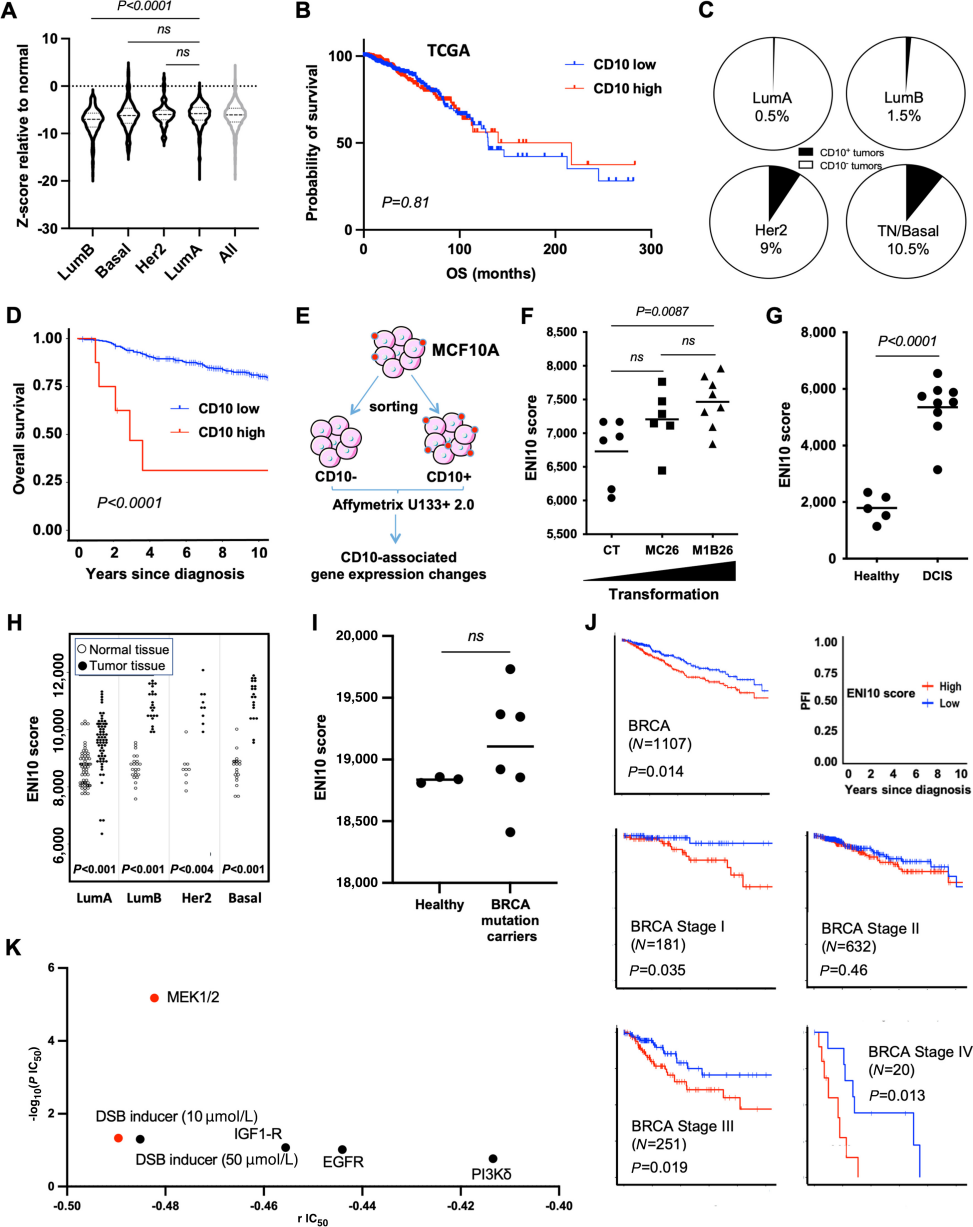
The molecular identity of untransformed CD10-positive cells increase with mammary gland transformation. **A,** CD10 transcript expression in TCGA breast tumors according to the molecular classification compared with normal samples. **B,** Kaplan–Meier plot of patients with breast cancers in TCGA cohort as a function of CD10 expression level. **C,** Quantification in a TMA of the percentage of tumors with CD10 IHC staining higher than 20% according to tumor subtype. **D,** Kaplan–Meier plot of patients with breast cancers from the TMA (as in C) as a function of CD10 protein expression level. **E,** Schematic representation of the experimental protocol used to obtain identify the ENI10 molecular signature. **F,** ssGSEA quantification of the CD10 signature score in the different MCF10A-derived cell lines with nontransformed (CT, *n* = 6), early (MC26, *n* = 6) and more aggressive (M1B26, *n* = 8) transformed models. **G,** ssGSEA ENI10 score of human primary DCIS versus healthy mammary gland (series GSE21422). **H,** ssGSEA ENI10 score of matched normal or tumoral breast tissue from breast cancer subtypes from TCGA's Pan-Cancer Atlas. **I,** ssGSEA ENI10 score from RNA-seq transcriptomic data from three normal breast epithelial cells samples (wildtype) and six BRCA mutated healthy epithelial cells samples. **J,** Kaplan–Meier plots of patients with breast cancers in TCGA cohort as a function of cancer stage and ENI10 score. **K,** Using transcriptomic data from the “Genomics of drug sensitivity in cancer” project from the Sanger Institute, ENI10 score of human breast cancers cell lines were correlated with their IC_50_ to 441 drugs. Drugs showing a significative negative correlation between the ENI10 ssGSEA score and IC_50_ (indicating a sensitivity to the drug when the ENI10 score increase) are shown. According to the GDSC guidelines, red dots show drugs with a significative IC_50_ correlation with the ENI10 score with a *P* value inferior to 0.001 and a Benjamini–Hochberg FDR inferior to 0.25. Black dots show suggestive correlations with a *P* value inferior to 0.005 and a nonparametric *P* value inferior to 0.1.

On the basis of the high expression of CD10 in our transformed models, we then checked CD10 expression within the breast cancer cohort of The Cancer Genome Atlas Program cohort (TCGA-BRCA; ref. 43). CD10 transcript levels were relatively stable among prediction analysis of microarray 50 (PAM50) subtypes in breast tumors and frequently under the level of expression observed in healthy tissue (Fig. 2A). Similarly, we observed no CD10 differential expression between breast cancer molecular subtypes in the Molecular Taxonomy of Breast Cancer International Consortium (METABRIC) cohort (ref. 44; Supplementary Fig. S1B). The CD10 transcript level was not predictive of patient outcome in TCGA-BRCA cohort (Fig. 2B) and high expression was associated with a marginally better survival in the METABRIC cohort (Supplementary Fig. S1C). We then evaluated CD10 protein expression in breast cancer by IHC staining in a breast tumor microarray (TMA). In total, only 2% of breast cancers of the different molecular subtypes tested (8/438 of the TMA) displayed a positive (≥20% CD10^+^ cells) intratumoral CD10 staining, while no significant difference between breast tumor subtypes was observed for CD10 staining in the stroma (Supplementary Table S2). The CD10^+^ tumors were mostly of the triple-negative molecular subtype that represented half of the CD10^+^ tumors (Fig. 2C). Finally, we observed a significant correlation between high CD10^+^ staining in tumors and poor OS of patients (Fig. 2D) that could be due to the poor prognosis of triple-negative tumors compared with other molecular subtypes.

While we uncovered an increased CD10 expression in our models of BMP2-driven transformation, no correlation was observed between CD10 mRNA expression in primary human breast cancers and prognosis. The pertinence of the correlation between CD10 protein expression and prognosis is reduced by the very low number of positive tumors. This suggests that the increase in CD10 expression could be specific to immature cells or SCs that are relatively scarce in primary tumors. This may have precluded any meaningful detection by bulk transcriptomic strategies. We wondered whether a molecular signature associated with CD10^+^ cells could be more easily detected and relevant in terms of patient prognosis. We performed a transcriptomic analysis of MCF10A cells sorted according to CD10 membrane expression (Fig. 2E), which led us to identify 159 genes upregulated in CD10^+^ compared with CD10^−^ cells (Supplementary Table S3). We called this molecular signature ENI10 (for early neoplasia index associated to CD10). We then measured ENI10 expression (called ENI10 score from now on) in our models of BMP2-driven transformation using ssGSEA and observed an increase in the ENI10 score between MCF10A-CT and M1B26 cells with an intermediate albeit not significantly different score for MC26 cells (Fig. 2F).

To assess the ENI10 score in early primary human breast transformation, we analyzed transcriptomic data from human ductal carcinoma *in situ* (DCIS) and normal breast tissue (45). This showed a significantly higher ENI10 score in DCIS versus healthy tissues (Fig. 2G). An increase in the ENI10 score was also observed in TCGA-BRCA dataset in each molecular breast cancer subtype compared with normal tissue (Fig. 2H). Interestingly, the ENI10 score was lower in the less aggressive Luminal A tumors compared with other subtypes. An increase in ENI10 score with breast tumor aggressiveness was also observed in the METABRIC dataset (Supplementary Fig. S1D).

In addition, we performed a RNA-seq analysis of human breast samples obtained from healthy donors undergoing esthetic surgery for breast size reduction or preventive mastectomies for BRCA-mutation carriers. Compared with mutation-free donors, breast tissue from BRCA-mutated carriers displayed an altered and variable ENI10 score indicating its potential value to follow pretransforming events (Fig. 2I). Considering the correlation between an increase in ENI10 score and breast cancer subtype aggressiveness, we evaluated the relationship between ENI10 score and patient survival in TCGA-BRCA dataset. A high ENI10 score was associated with a lower PFI in patients with breast cancer (Fig. 2J, first panel). The same observation was made in the METABRIC dataset (Supplementary Fig. S1E). Interestingly, when considering breast tumors according to stage, a high ENI10 score was also correlated with a lower PFI in early and late breast cancer stages (Fig. 2J). Nonetheless, the ENI10 score was not correlated to patient survival when breast tumors were stratified according to molecular subtypes, this suggests that in breast cancers the correlation between low ENI10 score and good prognosis could be due to the Luminal A tumors having both the lower ENI10 score and better prognosis (Supplementary Fig. S1F and S1G).

Next, using gene expression profiles from breast cancer cell lines included in the Cancer Cell Line Encyclopedia (46), we evaluated the correlation between the ENI10 score and the IC_50_ of 441 drugs already in clinical use or under development. The ENI10 score was correlated with response to several drugs, either indicative of resistance or sensitivity, depending on the drug. When focusing on drugs where the ENI10 score was inversely correlated with the IC_50_ (Supplementary Table S4), this analysis identified bleomycin, a drug that induces an arrest in the G_2_-phase of the cell cycle (47), as well as Refametinib (MEK inhibitor), as potential potent treatments for breast tumors that display a high CD10 score (Fig. 2K; Supplementary Table S5).

#### The ENI10 Molecular Signature is Enriched in Asymmetric Division-related Genes Controlled by CD10

**FIGURE 3:**
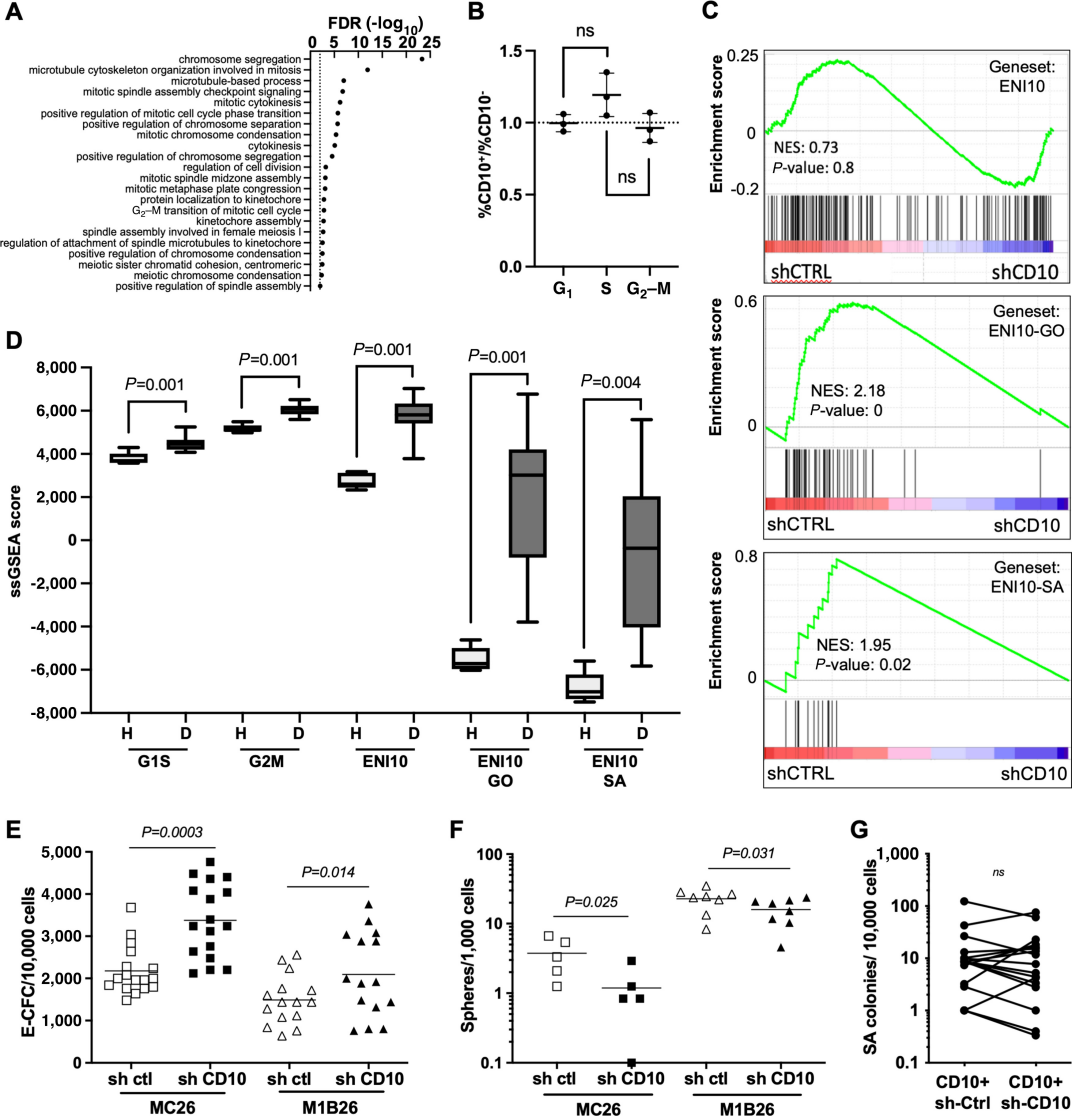
The ENI10 molecular signature is enriched in genes involved in chromosome segregation during mitosis. **A,** GO analysis on the CD10 signature genes using the “Biological Process” terms. The most downstream terms in the hierarchy with an FDR less than 0.01 are shown. **B,** Ratio of the percentage of CD10^+^ over CD10-negative cells in each phase of the cell cycle determined by flow-cytometry analysis of MCF10A-Fucci-CA cells stained with an anti-CD10 antibody. **C,** GSEA using RNA-seq experiments comparing MCF10A cells expressing a scramble (shCTRL) or CD10 specific shRNA (shCD10), the gene sets used are the ENI10 genes (top), ENI10 genes belonging to all enriched GO terms shown in A (middle) and ENI10 genes belonging to the “mitotic spindle assembly checkpoint signaling” GO term (bottom). **D,** ssGSEA quantification of the indicated gene sets in normal healthy breast tissue (H) or in DCIS (D) from GSE21422 series. **E,** Quantification of E-CFC from CD10^+^ MC26 or M1B26 cells infected with lentiviruses carrying a scramble (sh ctl) or sh CD10 vector. **F,** Quantification of spheres forming cells from CD10^+^ MC26 or M1B26 cells infected with lentiviruses carrying a scramble (sh ctl) or sh CD10 vector. **G,** Quantification of soft-agar clones from M1B26 CD10^+^ cells infected with lentiviruses carrying a scramble (sh ctl) or sh CD10 vector.

To gain insight into the functions enriched in the ENI10 molecular signature, we performed a GO analysis that revealed a strong enrichment in genes involved in the regulation of cell division, especially in the mechanistic control of chromosome condensation and segregation during the G_2_–M-phase (Fig. 3A). No significant enrichment in genes involved in S-phase was detected. A cell-cycle analysis of sorted CD10^+^ or CD10^−^ MCF10A cells revealed no imbalance in G_2_–M cells between the two populations, indicating that the enrichment in genes involved in G_2_–M molecular mechanisms in the ENI10 signature is not linked to cell-cycle status (Fig. 3B). The relationship between genes that define the ENI10 signature and the expression of CD10 itself was evaluated by knocking down CD10 expression using short interfering RNA (shRNA) in MCF10A cells (Supplementary Fig. S2A and S2B). No significant enrichment of the whole ENI10 signature in genes either upregulated or downregulated by the shCD10 was detected by GSEA (Fig. 3C, top). Interestingly, when we used as gene set in GSEA only the genes of the ENI10 signature belonging to the GO terms shown in Fig. 3A, we observed a strong enrichment in genes downregulated by the CD10 knockdown (Fig. 3C, middle). Of particular interest, a number of genes (*TKK1/MPS1, BUB1, BUB1B, AURKA, AURKB*) repressed in shCD10-MCF10A cells are known to play a role in the spindle assembly checkpoint (SAC). The SAC controls the asymmetric division mechanism and chromosome integrity/stability, and its dysregulation promotes aneuploidy and cancer (48, 49). When we used only the genes of the ENI10 signature belonging to the GO term “mitotic spindle assembly checkpoint signaling” shown in Fig. 3A for GSEA, we observed that all these genes were downregulated by CD10 knockdown (Fig. 3C, bottom). We then evaluated the contribution of genes involved in the G_2_–M-phase and in the SAC to the ability of the ENI10 signature to discriminate early transformed from normal tissues. As previously shown in Fig. 2G, the ENI10 ssGSEA score discriminated DCIS from healthy breast tissue. In that case, the variation of the ENI10 score is higher and therefore more discriminant than the ssGSEA score of genes known to be overexpressed in the G_1_–S or G_2_–M-phases of the cell cycle (Dominguez 2016), suggesting that the ability of ENI10 to distinguish between normal and transformed tissues is not solely a reflection of a higher proliferative state of the tumors. In addition, when restricting the ENI10 signature to genes belonging to the GO terms enriched in the signature or to the “mitotic spindle assembly checkpoint signaling” term, we observed a more stringent discrimination between healthy tissues and DCIS (Fig. 3D). This suggests that genes regulating the proper separation of the genetic material during mitosis could be specifically dysregulated in DCIS. Given the link between the SAC and asymmetric division as well as the importance of the latter in SC renewal, we tested the impact of CD10 knockdown on the SC population in our models. As MCF10A cells display immature properties similar to primary human mammary cells, we assessed the ability of MC26 and M1B26 cells to generate spheres and E-CFC (22, 37). Knocking down CD10 resulted in a significant increase in E-CFC frequency in both MC26 and M1B26 models illustrating their engagement in differentiation (Fig. 3E). Conversely, impairing CD10 expression significantly reduced the number of cell-forming spheres in MC26 and M1B26 cells (Fig. 3F). This indicates that, as for healthy mammary tissue (5), the CD10 protein is involved in the maintenance of stemness properties of transformed mammary epithelial cells. Interestingly, CD10 knockdown or overexpression did not significantly modify the ability of our cells to form soft-agar colonies, demonstrating that CD10 by itself is not required or sufficient for the maintenance or induction of the transformed phenotype (Fig. 3G; Supplementary Fig. S2A–S2F).

#### ENI10 Predicts Pan-Cancer Survival

**FIGURE 4:**
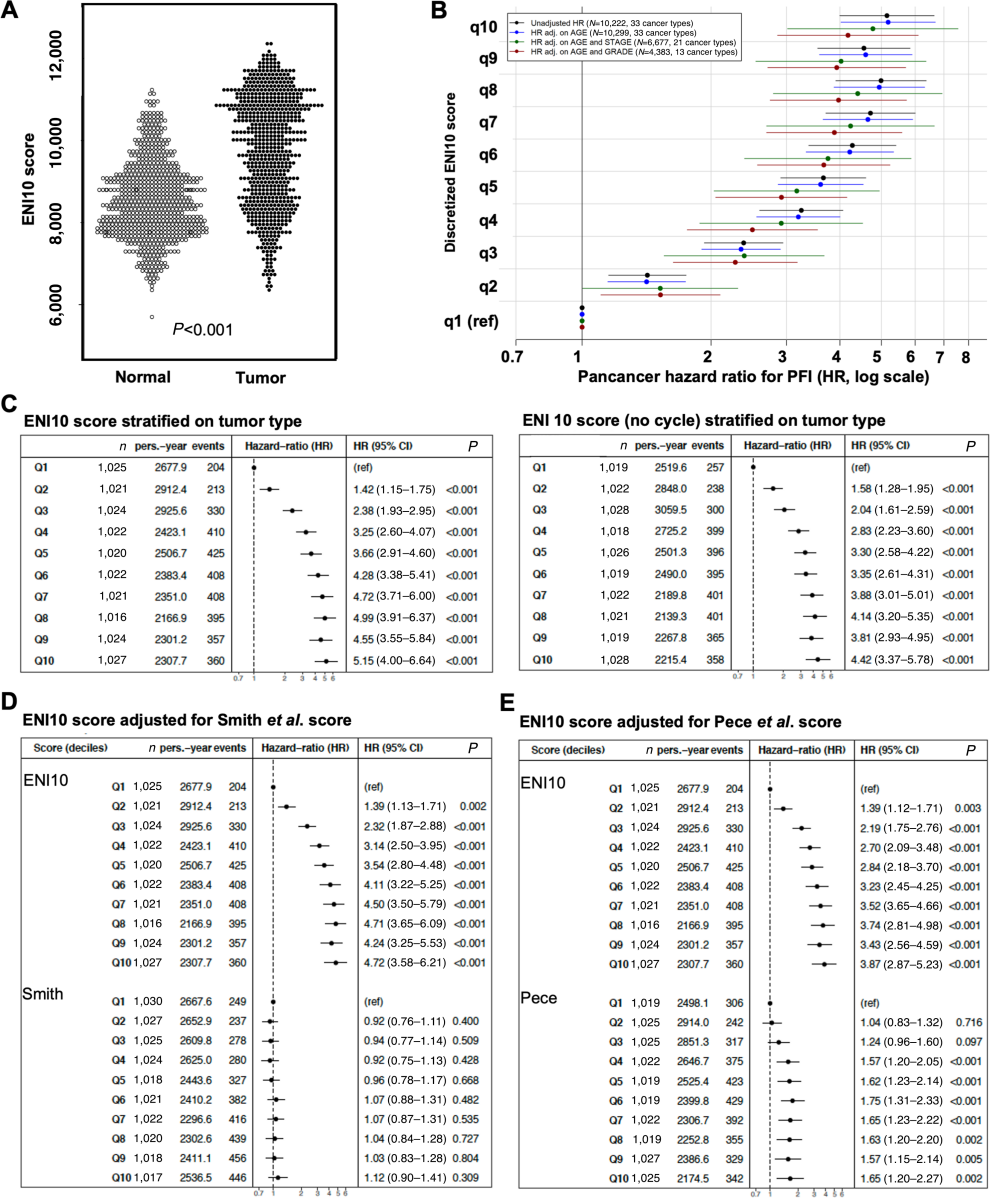
ENI10 predict pan-cancer survival independently of the cell cycle and more efficiently than other SC–derived signatures. **A,** ssGSEA score of the ENI10 signature in all tumors and normal samples from TCGA database. **B,** Correlation of CD10 signature score and survival in TCGA's Pan-Cancer Atlas. All tumor samples were pooled and the effect of the CD10-signature score discretized by deciles on survival outcome was evaluated from Cox models stratified on cancer types, using unadjusted (black marks), adjusted on age alone (blue marks, modeled with a 3-degree polynomial spline) or with a supplemental stratification term for stage (I/II/III/IV; green marks) or grade (1/2/3/4; red marks) pathologic scoring systems. Dots show the HR for PFI and adjacent bars the 95% confidence interval. **C,** Left: Overall Pan-Cancer analysis of the correlation between the ENI10 score and survival in TCGA's Pan-Cancer Atlas. All tumor samples were pooled and the effect of the ENI10 score discretized by deciles on survival outcome was evaluated from Cox models stratified on cancer types. Right: Same analysis with a reduced ENI10 signature were genes known to be regulated during the cell cycle were removed. **D** and **E,** Overall Pan-Cancer analysis of the effect of CD10 score on survival in TCGA's Pan-Cancer atlas. All tumor samples were pooled and the effect of the CD10 enrichment score discretized by deciles on survival outcome was evaluated using a multivariable Cox model including as covariables both the CD10 score and the Smith and colleagues (D) or Pece and colleagues (E) signatures*.* Dots show the hazard ratio for PFI and adjacent bars the 95% confidence interval.

Next, we quantified the ENI10 score in a large series of tumor samples from TCGA Pan-Cancer database (43). Our analyses showed a strong enrichment in the ENI10 score in tumor cells compared with non-tumor cells in a large range of tumors (>10,000 samples from 35 distinct solid tumors represented in this database), indicating a global association of ENI10 with the transformation status (Fig. 4A). To gain further insight into the prognostic value of ENI10 across tumor types, we adjusted multivariable stratified Cox models with a different baseline hazard for each tumor type. Remarkably, HRs adjusted according to age at diagnosis were almost identical to unadjusted HRs, and statistical adjustment based on stage or grade of disease did not alter the strong risk gradient (Fig. 4B). As the 159 genes used to compute the ENI10 score included a large number of genes associated with the G_2_–M-phases of the cell cycle (Fig. 3A), we further investigated the possibility that the ENI10 score could predict survival simply by measuring cell proliferation inside the tumor. To achieve this, we compared the predicted value of the ENI10 score using all of the 159 genes listed or an alternative ENI10 score excluding 25 genes shown to be upregulated in the G_2_–M-phases (ref. 50; *TTK*, *FAM64A*, *NUSAP1*, *BUB1*, *PRC1*, *CDC25C*, *SPAG5*, *CCNA2*, *TOP2A*, *ESPL1*, *CCNF*, *BUB1B*, *CCNB1*, *KIF2C*, *HMMR*, *UBE2C*, *CENPE*, *KPNA2*, *CENPF*, *CDCA3*, *TACC3*, *KIF23*, *MKI67*, *NEK2*, *HMGB2*). This analysis indicated that this alternative ENI10 score remained similarly predictive of patient survival (Fig. 4C).

Next, we tested the added predictive value of the ENI10 over two previously identified signatures obtained from healthy adult tissue SCs (51, 52). These two studies aimed at understanding the relationship between epithelial cancers and SC transcriptional programs using, as in our present study, epithelial SCs as a starting point. Pece and colleagues identified a CSC molecular signature of 20 genes specifically expressed in normal epithelial mammary SCs (51). Smith and colleagues used a pan-SC and Pan-Cancer approach to identify a transcriptional signature shared by epithelial adult normal SCs and tumors (52) and isolated a signature consisting of the top 50 genes associated with adult SCs, naive or primed human embryonic SCs, with no gene overlap among the three SC signatures. To investigate the added value of ENI10 compared with these two other signatures, we calculated their respective score by ssGSEA and fitted Cox models for the ENI10 score, including the two other scores as adjustment variables. The ENI10 score, which represents the molecular signature of premalignant SCs, displayed high HRs when adjusted against the Smith (ref. 52; Fig. 4D) or Pece (ref. 51; Fig. 4E) cancer and SC-related scores. Of note, after adjustment against the ENI10 score, the predictive value of the Pece and colleagues score was markedly reduced and the Smith and colleagues score completely lost statistical significance. These findings indicate that the ENI10 score is a more robust and powerful way to predict clinical outcomes in many different solid tumors than signatures of normal and CSCs.

#### ENI10 is a Robust and Independent Prognostic Factor for Several Solid Tumors and to Screen Drugs

**FIGURE 5:**
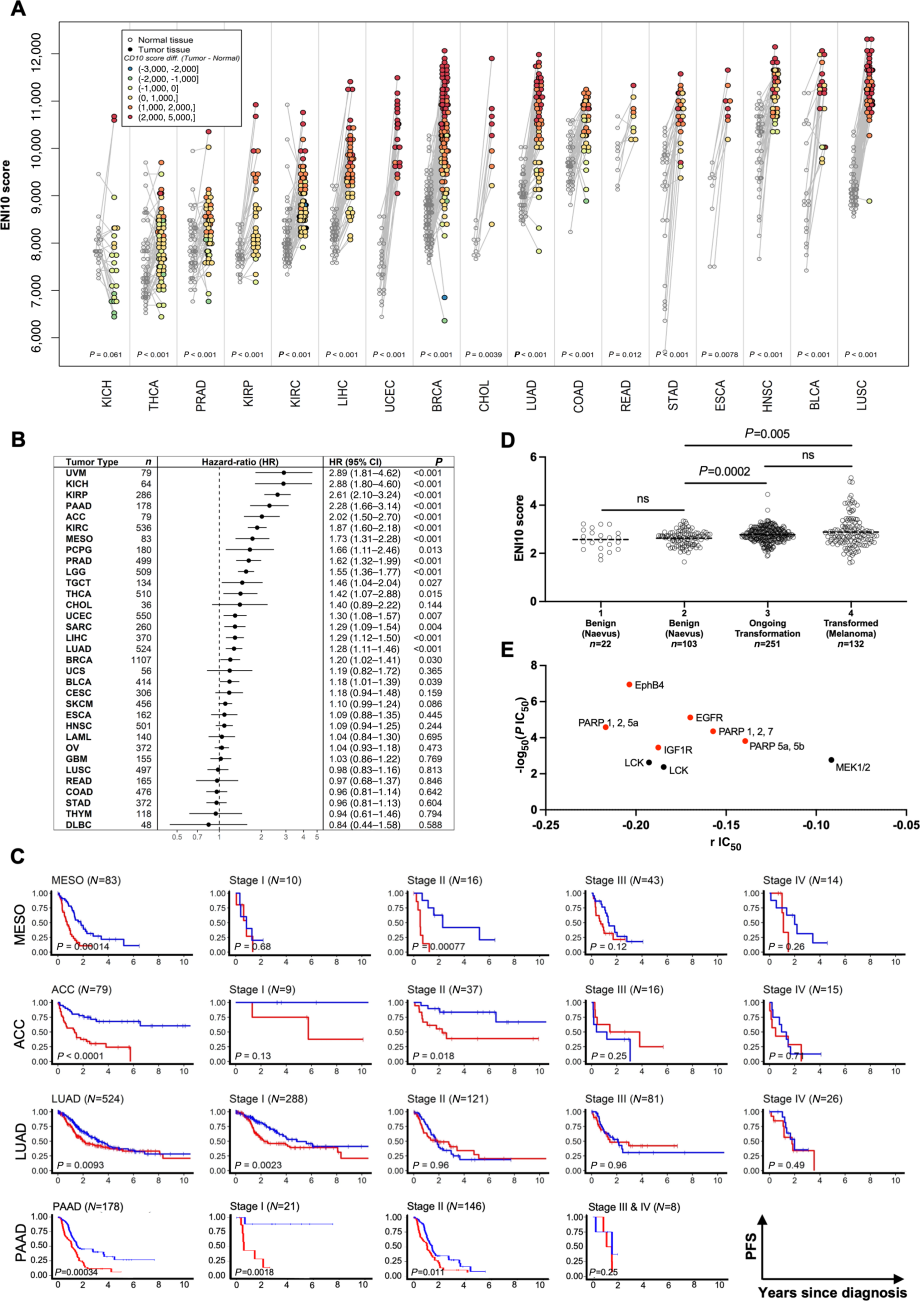
An increased ENI10 score predicts patient's survival in several cancer types. **A,** ssGSEA ENI10 score in pairs of normal and tumor samples from TCGA's Pan-Cancer atlas linked by gray lines and the difference are color coded on the dots representing the tumor samples. ACC: adrenocortical carcinoma, BLCA: bladder urothelial carcinoma, BRCA: breast invasive carcinoma, CESC: cervical squamous cell carcinoma and endocervical adenocarcinoma, CHOL: cholangiocarcinoma, COAD: colon adenocarcinoma, DLBC: lymphoid neoplasm diffuse large B-cell lymphoma, ESCA: esophageal carcinoma, HNSC: head and neck squamous cell carcinoma, KICH: kidney chromophobe, KIRC: kidney renal clear cell carcinoma, KIRP: kidney renal papillary cell carcinoma, LAML: acute myeloid leukemia, LGG: low-grade glioma, LIHC: liver hepatocellular carcinoma, LUAD: lung adenocarcinoma, LUSC: lung squamous cell carcinoma, MESO: mesothelioma, OV: ovarian cancer, PCPG: pheochromocytoma and paraganglioma, PRAD: prostate adenocarcinoma, READ: rectum adenocarcinoma, SARC: sarcoma, SKCM: skin cutaneous melanoma, STAD: stomach adenocarcinoma, TGCT: testicular germ cell tumor, THCA: thyroid carcinoma, THYM: thymoma, UCEC: uterine corpus endometrial carcinoma, UCS: uterine carcinosarcoma, UVM: uveal melanoma. **B,** Correlation of the ENI10 score and survival outcome for each type of cancer of TCGA Pan-Cancer atlas estimated by HRs of PFS corresponding to one SD of the score taken as a continuous variable. Dots show the HR and adjacent bars the 95% confidence interval. **C,** Examples of PFS curves from TCGA Pan-Cancer atlas in the whole cohort and as a function of stage estimated using the Kaplan–Meier method and compared with the log-rank test between groups of patients defined by the median of the ENI10 score (low scores in blue and high scores in red). **D,** ssGSEA ENI10 score in transcriptomic data from nevus or melanoma at different stage of clinically defined transformation. **E,** Using transcriptomic data from the “Genomics of drug sensitivity in cancer” project from the Sanger Institute, ENI10 score of all human cancers cell lines available were correlated with their IC_50_ to 441 drugs. Targets of the drugs with a significative negative correlation between the ENI10 ssGSEA score and IC_50_ (indicating a sensitivity to the drug when the ENI10 score increase) are shown. According to the GDSC guidelines, red dots show drugs with a significative IC_50_ correlation with the ENI10 score with a *P* value inferior to 0.001 and a Benjamini–Hochberg FDR inferior to 0.25. Black dots show suggestive correlations with a *P* value inferior to 0.005 and a nonparametric *P* value inferior to 0.1.

Because using the pan-cancer strategy shown in Fig. 4, it can be difficult to test all confounding variables, especially cancer type, due to the high number of samples required to use the decile approach, we then evaluated CD10 expression as well as the association of the ENI10 signature with the transformation at the level of individual solid tumors represented in TCGA database. At the transcript level, there was no clear CD10 dysregulation compared with normal tissues in any TCGA cancer type (Supplementary Fig. S3A). Moreover, data from the Human Protein Atlas (53) showed that at the protein level, CD10 is only detectable in a fraction of cancer types (Supplementary Fig. S3B). On the other hand, in all cancer types but one where matched normal tissues were available, we observed a significant increase in the ENI10 score in tumors (Fig. 5A). We next analyzed the ability of the ENI10 score to predict patient survival in 33 different cancers of TCGA database. Analyses by Cox regression models highlighted that the CD10 score is associated with poor PFI for more than 50% (18/33) of the tested cancer types (Fig. 5B). Kaplan–Meier curves for all cancer types where the ENI10 score predicted survival are shown (Supplementary Fig. S3C). Importantly, when available we analyzed the predictive value of the ENI10 score as a function of tumor stage. This analysis revealed that in a number of cancers, the ENI10 score discriminates patients with a worse prognosis even at an early stage, including for the very aggressive pancreatic adenocarcinoma (Fig. 5C). Patient survival was the most strongly determined by the ENI10 score for TCGA uveal melanoma (UVM; Supplementary Fig. S3C). Because UVM is a rare and very specific type of melanoma, we explored an in-house cohort of skin nevus or melanoma at different stages of clinically-defined transformation. As observed in breast tissue, the ENI10 score increased within tumor cells even at very early stages of skin transformation (Fig. 5D). The same increase in ENI10 score was found using transcriptomic data from another cohort of benign melanocytic nevi and primary melanoma from the literature (ref. 54; Supplementary Fig. S3D).

We then attempted to correlate the ENI10 score and drug response using all cancer types represented in the Cancer Cell Line Encyclopedia. A subset of 9 drugs appeared to efficiently target a broad range of cancer cell lines expressing high levels of the ENI10 score (Fig. 5E; Supplementary Tables S5 and S6). This included cetuximab (as in the breast cancer specific analysis), IGF1R and LCK inhibitors, all identified to modulate the SAC (55, 56). Very interestingly, three of the nine drugs are inhibitors of PARP also reported to downregulate the SAC and induce a G_2_–M arrest (57).

Collectively, these findings indicate that using the ENI10 molecular signature is a robust and powerful way to predict clinical outcome in a large number of different solid tumors. In addition, our analyses unveiled a short list of drugs that may efficiently target cancer cells with a high ENI10 score, likely owing to their ability to modulate SAC-related elements. Altogether, these data strongly suggest that the ENI10 signature may help to identify high-risk patients and tailor systemic therapy in patients with cancer.

## Discussion

We explored the importance of CD10 expression during mammary SC transformation using a new series of breast cancer models based on non–oncogene-driven transformation of the MCF10A cell line that we developed by chronic exposure to BMP2 (21). We unveil that CD10 expression increases with cell transformation and remains linked to SC-like properties in fully transformed cells, though it was not necessary to maintain a transformed state. This is consistent with data reported in breast cancer (7), melanoma (58), lung cancer, mesothelioma (59, 60), or head and neck squamous cell carcinoma (6), and indicates that CD10^+^ cells share common features with SC both in their normal and transformed state. We extracted a CD10^+^ SC-specific molecular signature of 159 genes enriched in primary breast cancers and identified this ENI10 index as a reliable marker for breast cancer prognosis (44, 45).

The ENI10 was significantly enriched in various solid tumor tissues compared with paired healthy tissues regardless of the initial ENI10 level. In addition, using our breast cancer MCF10A-derived BMP2-driven early transformation model or primary non-tumoral tissues from BRCA-mutated carriers, we established that the ENI10 is a powerful tool to identify very early transformation processes, further confirmed in the context of melanoma in which CD10 has been associated with aggressiveness and treatment escape (58). The increased risk gradient observed in a Pan-Cancer Cox model, highlighted a dose–response relationship of the effect of the ENI10 on patient outcome. A role for BMP signaling has been reported in cancers for which we identify that a high ENI10 was predictive of poor prognosis [melanoma (61, 62), lung adenocarcinoma (63), Glioma (64, 65), clear renal carcinoma (66), prostate (67, 68) or pancreas (69, 70)]. In addition, a direct link between CD10-expressing cells and a BMP-SC response is described in lymphoid (17), breast (38) or nervous system (18) as well as during cancer formation or progression (23). Altogether, it suggests that within CD10-expressing immature cells a cellular subset could constitute a preferential target of the transformation process which will consequently lead to an enrichment of the ENI10. Therefore, CD10^+^ cells could constitute a preferential pool of cells highly sensitive to a BMP-driven transformation (70). Modulation of CD10 expression confirmed its direct control of a significant number of genes of the ENI10 involved in G_2_–M, such as the SAC. Importantly, SAC-related genes are involved in asymmetric cell division, a key SC feature (71, 72) that allows one of the two daughter cells to preferentially inherit the leading strand (mother) chromatid (73). This ensures fidelity of chromosome segregation and prevents chromosome instability. Dysregulation of the SAC promote aneuploidy, tumor initiation, and progression (48, 49). Interestingly, in breast cancer cells, BMP signaling controls genes of the mitotic checkpoints of the SAC (*TTK/MPS1*; ref. 74), highlighting a link between BMP-responsive SCs, CD10 and the asymmetric division process ensured by SAC-related genes. It suggests a role for CD10 in preventing the acquisition of chromosomal instability by SC that could contribute to resistance and maintenance of CD10-expressing CSC. Also, drugs that induce a G_2_–M arrest and target the SAC, such as PARP inhibitors (PARPi; ref. 57), cetuximab (75), IGF1R, and LCK inhibitors (46, 55, 56), seemed to particularly predict efficiency against cancer cells with a high ENI10 score. In this context, pancreatic cancer (PAAD) is especially impressive as the ENI10 score identify patients with PAAD at early stages or grades that could benefit from PARPis as suggested (76).

In summary, we identified a molecular signature related to the CD10 function on SC features and representative of premalignant cells even though CD10 itself does not drive cell transformation. This ENI10 is linked to cancer evolution and patient survival and may contribute to identifying effective therapies. This score appears to be unique, powerful and highly robust to help predict cancer evolution in many different cancer types including very early stages of the disease in the worst types of solid cancers. Further analysis in various clinical settings, for example, focusing on specific cancers like PAAD or response to treatments like PARPi could lead to define in each case clinically useful thresholds of the ENI10 score for patient management.

## Supplementary Material

Supplementary Figure S1 Supplementary Figure S1 shows the doubling time of MCF10A-CT, MC26 and M1B26 cell lines in A; the expression of the CD10 mRNA in the METABRIC cohort in B; the probability of survival of patients from the METABRIC cohort in function of CD10 expression in C; the ENI10 score of patients form the METABRIC cohort in D; the relapse-free interval of patients from the METABRIC cohort in function of the ENI10 score in E and the progression free interval in F or overall survival in G of patients form the METABRIC cohort in function of the ENI10 score and of the molecular subtype of the breast tumor.

Supplementary Figure S2 Supplementary Figure S2 shows in A the percentage of indicated cells positive for CD10 staining by flow-cytometry and in B the mean fluorescence intensity of CD10-positive cells after infection with a vector expressing a control or anti-CD10 shRNA. In C the percentage of indicated cells positive for CD10 staining by flow-cytometry and in D the mean fluorescence intensity of CD10-positive cells after infection with a vector expressing the CD10 cDNA or an empty control vector are shown. In E the frequency of soft-agar colony forming cells is shown in the indicated cell lines after infection with a vector expressing the CD10 cDNA or an empty control vector. In F the frequency of soft-agar colony forming cells is shown in the indicated cell lines after infection with a vector expressing a control or anti-CD10 shRNA.

Supplementary Figure S3 Supplementary Figure S3 shows in A the CD10 transcript expression level in various cancer types from the TCGA cohorts. B shows the expression level of the CD10 protein in various cancer types from the human protein atlas. C shows the progression free intervals in function of the ENI10 score of various cancer types from the TCGA cohorts. D shows the ENI10 score of cohorts of benign melanocytic nevi or primary melanoma.

Supplementary Table S1 Supplementary Table S1 shows the full Gene Ontology terms enrichment analysis of the indicated cell lines transcriptome.

Supplementary Table S2 Supplementary Table S2 shows the results of the CD10 IHC staining on a breast cancer tumor micro-array.

Supplementary Table S3 Supplementary Table S3 shows the genes part of the ENI10, ENI10-GO and ENI10-SA molecular signatures.

Supplementary Table S4 Supplementary Table S4 shows the correlation between the ENI10 score and the IC50 of the indicated drugs in breast cancer cell lines from the "Genomics of Drug Sensitivity in Cancer Project".

Supplementary Table S5 Supplementary Table S5 shows the drugs corresponding to the targets indicated in Figures 2K and 5E of the main text.

Supplementary Table S6 Supplementary Table S6 shows the correlation between the ENI10 score and the IC50 of the indicated drugs in cancer cell lines from the "Genomics of Drug Sensitivity in Cancer Project".
