# Supplementary material for: An Early Neoplasia Index (ENI10), Based on Molecular Identity of CD10 Cells and Associated Stemness Biomarkers, is a Predictor of Patient Outcome in Many Cancers
Source: Cancer Res Commun. 2023 Sep 29;3(9):1966–80. doi: 10.1158/2767-9764.CRC-23-0196 (PMC10540743; doi:10.1158/2767-9764.CRC-23-0196)
Supplement: Supplementary Figure S1 — shows the doubling time of MCF10A-CT, MC26 and M1B26 cell lines in A; the expression of the CD10 mRNA in the METABRIC cohort in B; the probability of survival of patients from the METABRIC cohort in function of CD10 expression in C; the ENI10 score of patients form the METABRIC cohort in D; the relapse-free interval of patients from the METABRIC cohort in function of the ENI10 score in E and the progression free interval in F or overall survival in G of patients form the METABRIC cohort in function of the ENI10 score and of the molecular subtype of the breast tumor. [file crc-23-0196-s01.pdf]

Supplementary Figure S1

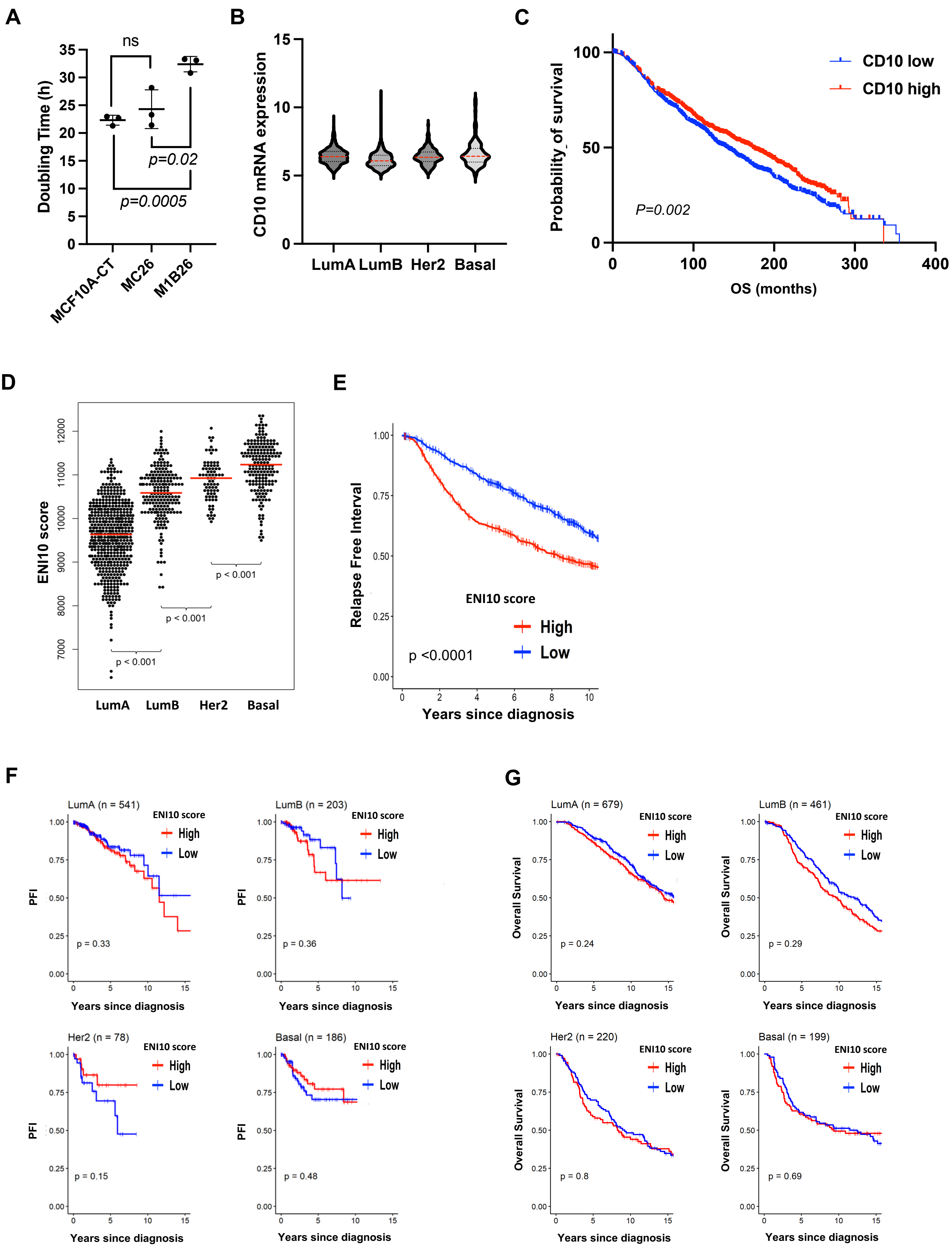

Supplementary Figure S1. A, Doubling time in hours of the indicated cell lines. Three independent replicates are shown with the mean and standard deviation. B, CD10 transcript expression in the breast tumors of the METABRIC database according to molecular subtypes. C, Kaplan-Meier plot of breast cancers patients in the METABRIC cohort as a function of CD10 expression level. D, ssGSEA ENI10 score of breast tumor according to cancer molecular subtypes from the METABRIC database. E, Kaplan-Meier plot of breast cancers patients in the METABRIC cohort divided by the median of the ENI10 score (low scores in blue and high scores in red). F, Kaplan-Meier plot of breast cancers patients in the TCGA-BRCA cohort according to the ENI10 score (as in E) and the molecular subtypes. G, Kaplan-Meier plot of breast cancers patients in the METABRIC cohort according to the ENI10 score (as in E) and the molecular subtypes.
