# Supplementary material for: An Early Neoplasia Index (ENI10), Based on Molecular Identity of CD10 Cells and Associated Stemness Biomarkers, is a Predictor of Patient Outcome in Many Cancers
Source: Cancer Res Commun. 2023 Sep 29;3(9):1966–80. doi: 10.1158/2767-9764.CRC-23-0196 (PMC10540743; doi:10.1158/2767-9764.CRC-23-0196)
Supplement: Supplementary Figure S2 — shows in A the percentage of indicated cells positive for CD10 staining by flow-cytometry and in B the mean fluorescence intensity of CD10-positive cells after infection with a vector expressing a control or anti-CD10 shRNA. In C the percentage of indicated cells positive for CD10 staining by flow-cytometry and in D the mean fluorescence intensity of CD10-positive cells after infection with a vector expressing the CD10 cDNA or an empty control vector are shown. In E the frequency of soft-agar colony forming cells is shown in the indicated cell lines after infection with a vector expressing the CD10 cDNA or an empty control vector. In F the frequency of soft-agar colony forming cells is shown in the indicated cell lines after infection with a vector expressing a control or anti-CD10 shRNA. [file crc-23-0196-s02.pdf]

Supplementary Figure S2

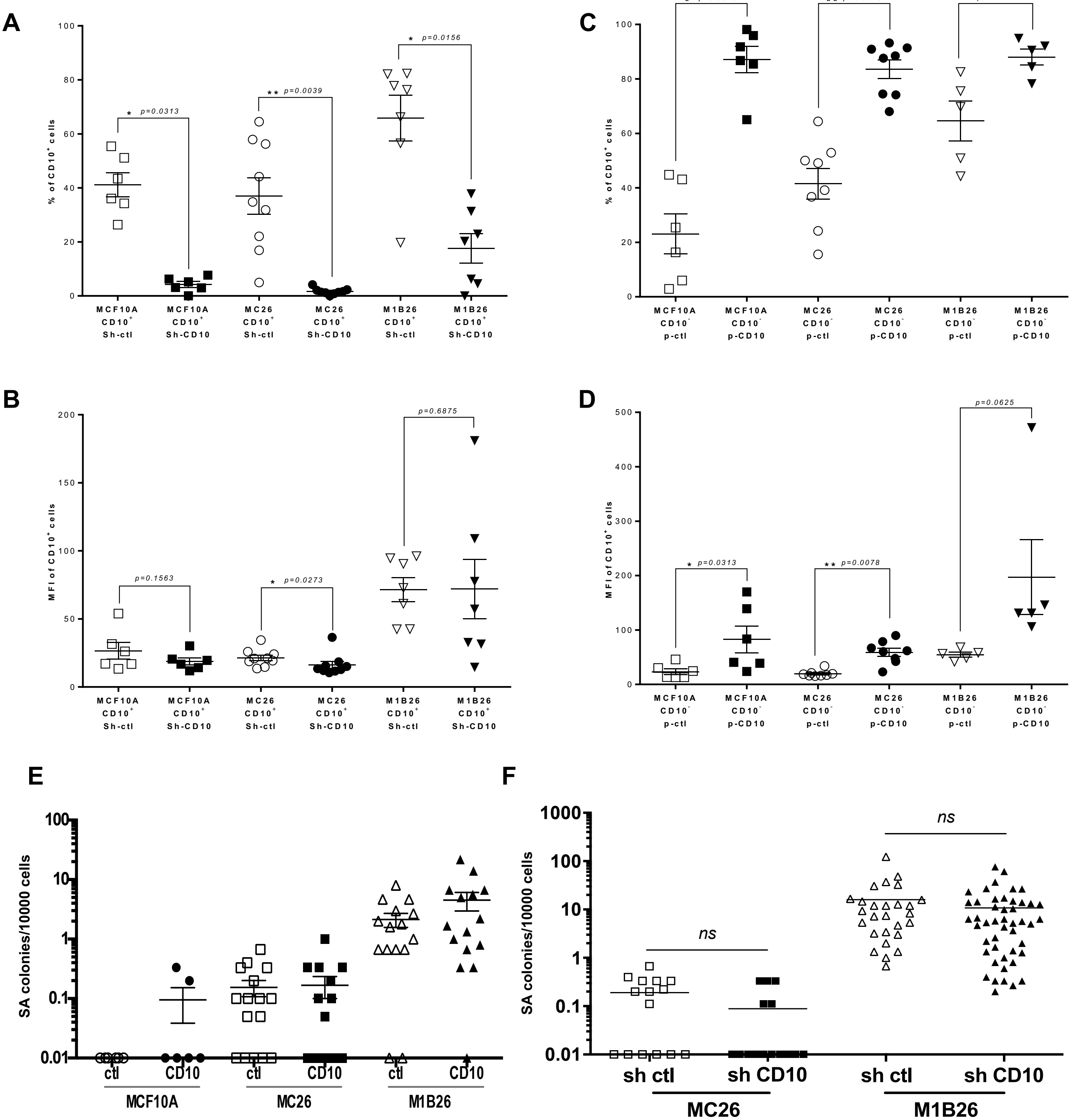

Supplementary Figure S2. A,B, FACS sorted CD10$^+$ MCF10A-CT (squares), MC26 (circles) or M1B26 (triangles) cells were transduced with a lentiviral vector expressing a shRNA targeting CD10 (sh-CD10, black symbols) or a control shRNA (sh-ctl, open symbols). CD10 membrane expression was then measured by flow-cytometry and results shown as percentage of CD10$^+$ cells (A) or CD10 MFI (mean fluorescence intensity) of CD10$^+$ cells (B). C, D, FACS sorted CD10$^-$ MCF10A-CT (squares), MC26 (circles) or M1B26 (triangles) cells were transduced with a lentiviral CD10 expression vector (p-CD10, black symbols) or a control, empty vector (p-ctl, open symbols). CD10 membrane expression was then measured by flow-cytometry and results shown as percentage of CD10$^+$ cells (C) or CD10 MFI (mean fluorescence intensity) of CD10$^+$ cells (D). E, Soft-agar colony formation assay with sorted CD10$^-$ MCF10A-CT, MC26 and M1B26 cells infected by lentiviruses carrying an empty (ctl) or CD10 expression vector. F, Soft-agar colony formation assay with MC26 (n=21) or M1B26 (n=29) cells expressing a scramble (sh ctl) or CD10 specific shRNA.
