# Supplementary material for: An Early Neoplasia Index (ENI10), Based on Molecular Identity of CD10 Cells and Associated Stemness Biomarkers, is a Predictor of Patient Outcome in Many Cancers
Source: Cancer Res Commun. 2023 Sep 29;3(9):1966–80. doi: 10.1158/2767-9764.CRC-23-0196 (PMC10540743; doi:10.1158/2767-9764.CRC-23-0196)
Supplement: Supplementary Figure S3 — shows in A the CD10 transcript expression level in various cancer types from the TCGA cohorts. B shows the expression level of the CD10 protein in various cancer types from the human protein atlas. C shows the progression free intervals in function of the ENI10 score of various cancer types from the TCGA cohorts. D shows the ENI10 score of cohorts of benign melanocytic nevi or primary melanoma. [file crc-23-0196-s03.pdf]

Supplementary Figure S3

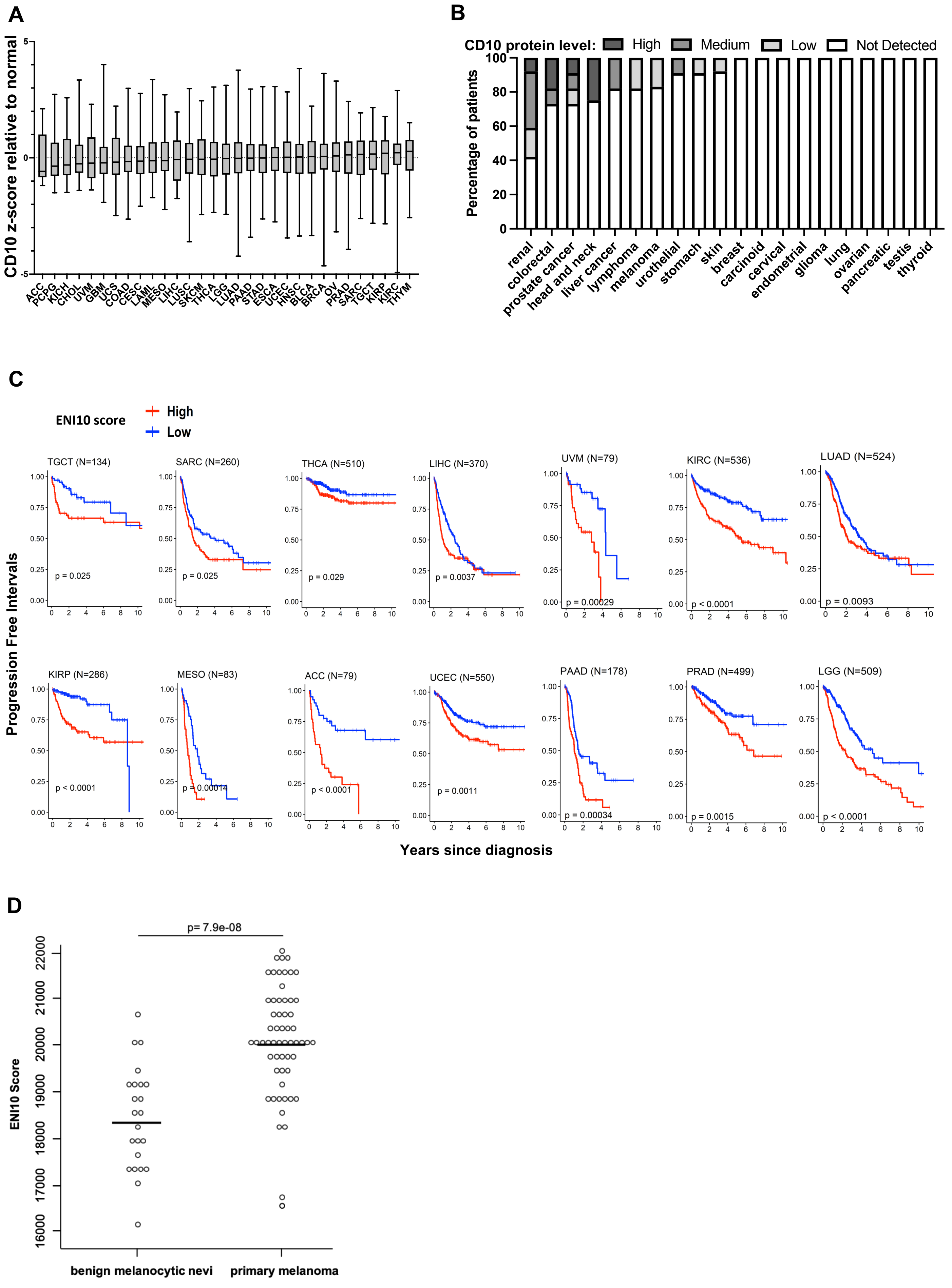

Supplementary Figure S3. A, CD10 transcript expression in TCGA tumor types relative to normal samples. B, CD10 protein expression in tumor types from the human protein atlas. C, Kaplan-Meier plot of various cancers patients in the TCGA cohorts divided by the median of the ENI10 score (low scores in blue and high scores in red). D, ssGSEA ENI10 score in transcriptomic data (GSE112509) from benign melanocytic nevi or primary melanoma (47). Each individual sample is indicated by an open circle and the median is shown as a bar.
