# Supplementary material for: An Early Neoplasia Index (ENI10), Based on Molecular Identity of CD10 Cells and Associated Stemness Biomarkers, is a Predictor of Patient Outcome in Many Cancers
Source: Cancer Res Commun. 2023 Sep 29;3(9):1966–80. doi: 10.1158/2767-9764.CRC-23-0196 (PMC10540743; doi:10.1158/2767-9764.CRC-23-0196)
Supplement: Supplementary Table S1 — shows the full Gene Ontology terms enrichment analysis of the indicated cell lines transcriptome. [file crc-23-0196-s04.pdf]

Supplementary Table S1. GO term enrichment analysis comparing MC26 and M1B26 to MCF10A-CT cells.

MC26		M1B26		MC26/M1B26	Colonne3
GO term	p-value	GO term	p-value	GO term	p-value
developmental process (GO:0032502)	5,68E-08	DNA replication (GO:0006260)	2,65E-07	multicellular organismal process (GO:0032501)	1,22E-08
multicellular organism development (GO:0007275)	1,62E-07	negative regulation of cellular process (GO:0048523)	7,24E-07	developmental process (GO:0032502)	2,67E-08
tissue development (GO:0009888)	1,70E-07	cell cycle G1/S phase transition (GO:0044843)	7,89E-07	multicellular organism development (GO:0007275)	6,75E-08
anatomical structure development (GO:0048856)	1,76E-07	response to stimulus (GO:0050896)	8,80E-07	anatomical structure development (GO:0048856)	7,18E-08
regulation of developmental process (GO:0050793)	2,32E-07	G1/S transition of mitotic cell cycle (GO:0000082)	8,87E-07	regulation of signaling (GO:0023051)	1,03E-07
anatomical structure morphogenesis (GO:0009653)	2,37E-07	response to stress (GO:0006950)	8,88E-07	regulation of cell communication (GO:0010646)	1,07E-07
regulation of cell proliferation (GO:0042127)	6,79E-07	mitotic cell cycle phase transition (GO:0044772)	1,05E-06	regulation of response to stimulus (GO:0048583)	1,15E-07
system development (GO:0048731)	1,10E-06	DNA-dependent DNA replication (GO:0006261)	1,18E-06	response to organic substance (GO:0010033)	2,16E-07
negative regulation of biological process (GO:0048519)	1,17E-05	anatomical structure development (GO:0048856)	1,27E-06	regulation of signal transduction (GO:0009966)	3,07E-07
negative regulation of cell proliferation (GO:0008285)	1,64E-05	system development (GO:0048731)	1,37E-06	system development (GO:0048731)	3,29E-07
regulation of multicellular organismal development (GO:2000026)	6,30E-05	negative regulation of biological process (GO:0048519)	1,45E-06	regulation of multicellular organismal process (GO:0051239)	9,94E-07
negative regulation of cellular process (GO:0048523)	6,39E-05	multicellular organism development (GO:0007275)	1,49E-06	response to wounding (GO:0009611)	2,63E-06
cellular developmental process (GO:0048869)	9,14E-05	cell cycle phase transition (GO:0044770)	1,54E-06	negative regulation of cellular process (GO:0048523)	6,92E-06
regulation of multicellular organismal process (GO:0051239)	9,50E-05	cellular response to chemical stimulus (GO:0070887)	2,56E-06	regulation of anatomical structure morphogenesis (GO:0022603)	7,03E-06
epithelium development (GO:0060429)	9,59E-05	response to organic substance (GO:0010033)	2,92E-06	regulation of proteolysis (GO:0030162)	7,11E-06
cell differentiation (GO:0030154)	9,85E-05	developmental process (GO:0032502)	4,80E-06	positive regulation of biological process (GO:0048518)	7,18E-06
multicellular organismal process (GO:0032501)	1,27E-04	blood vessel morphogenesis (GO:0048514)	5,02E-06	Unclassified (UNCLASSIFIED)	1,36E-05
animal organ development (GO:0048513)	1,28E-04	angiogenesis (GO:0001525)	8,77E-06	biological_process (GO:0008150)	1,44E-05
extracellular matrix organization (GO:0030198)	2,23E-04	response to chemical (GO:0042221)	9,27E-06	regulation of biological quality (GO:0065008)	1,51E-05
response to stress (GO:0006950)	2,34E-04	cellular response to organic substance (GO:0071310)	9,97E-06	response to chemical (GO:0042221)	2,74E-05
biological regulation (GO:0065007)	2,77E-04	DNA replication initiation (GO:0006270)	1,17E-05	regulation of multicellular organismal development (GO:2000026)	3,48E-05
regulation of biological process (GO:0050789)	3,18E-04	response to cytokine (GO:0034097)	1,69E-05	tissue development (GO:0009888)	3,65E-05
extracellular structure organization (GO:0043062)	3,25E-04	biological regulation (GO:0065007)	2,35E-05	wound healing (GO:0042060)	4,41E-05
cellular process (GO:0009987)	4,04E-04	cellular process (GO:0009987)	3,98E-05	negative regulation of biological process (GO:0048519)	5,12E-05
regulation of cellular process (GO:0050794)	6,00E-04	positive regulation of multicellular organismal process (GO:0051240)	4,12E-05	tube morphogenesis (GO:0035239)	5,44E-05
regulation of cellular component biogenesis (GO:0044087)	8,29E-04	anatomical structure formation involved in morphogenesis (GO:0048646)	4,13E-05	regulation of developmental process (GO:0050793)	5,55E-05
regulation of cell adhesion (GO:0030155)	8,30E-04	anatomical structure morphogenesis (GO:0009653)	4,50E-05	secretion by cell (GO:0032940)	5,93E-05
regulation of cellular component organization (GO:0051128)	8,76E-04	positive regulation of angiogenesis (GO:0045766)	4,56E-05	regulation of vasculature development (GO:1901342)	6,42E-05
positive regulation of biological process (GO:0048518)	9,40E-04	positive regulation of developmental process (GO:0051094)	6,33E-05	biological regulation (GO:0065007)	7,21E-05
positive regulation of cellular process (GO:0048522)	9,68E-04	tube morphogenesis (GO:0035239)	6,91E-05	anatomical structure formation involved in morphogenesis (GO:0048646)	9,70E-05
		tube development (GO:0035295)	8,07E-05	regulation of cell differentiation (GO:0045595)	9,80E-05
		response to external stimulus (GO:0009605)	8,08E-05	cellular response to chemical stimulus (GO:0070887)	1,01E-04
		blood vessel development (GO:0001568)	8,12E-05	positive regulation of cellular process (GO:0048522)	1,34E-04
		multicellular organismal process (GO:0032501)	8,22E-05	cellular developmental process (GO:0048869)	1,36E-04
		positive regulation of vasculature development (GO:1904018)	1,10E-04	regulation of cell proliferation (GO:0042127)	1,48E-04
		regulation of response to stimulus (GO:0048583)	1,13E-04	positive regulation of signal transduction (GO:0009967)	1,54E-04
		vasculature development (GO:0001944)	1,64E-04	negative regulation of multicellular organismal process (GO:0051241)	1,55E-04
		response to abiotic stimulus (GO:0009628)	1,77E-04	positive regulation of cell communication (GO:0010647)	1,56E-04
		regulation of localization (GO:0032879)	1,79E-04	cell differentiation (GO:0030154)	1,56E-04
		regulation of locomotion (GO:0040012)	2,00E-04	blood vessel development (GO:0001568)	1,58E-04
		cardiovascular system development (GO:0072358)	2,01E-04	positive regulation of signaling (GO:0023056)	1,63E-04
		mitotic cell cycle process (GO:1903047)	2,01E-04	positive regulation of apoptotic process (GO:0043065)	1,69E-04
		DNA metabolic process (GO:0006259)	2,07E-04	angiogenesis (GO:0001525)	1,71E-04
		positive regulation of cellular process (GO:0048522)	2,40E-04	positive regulation of programmed cell death (GO:0043068)	1,85E-04
		regulation of cell migration (GO:0030334)	2,41E-04	regulation of cell migration (GO:0030334)	1,85E-04
		cellular response to cytokine stimulus (GO:0071345)	2,71E-04	response to oxygen-containing compound (GO:1901700)	1,86E-04
		regulation of catalytic activity (GO:0050790)	2,72E-04	regulation of cellular component movement (GO:0051270)	1,87E-04
		regulation of cell motility (GO:2000145)	2,73E-04	epithelium development (GO:0060429)	1,88E-04
		animal organ development (GO:0048513)	2,75E-04	secretion (GO:0046903)	2,00E-04
		immune system process (GO:0002376)	3,16E-04	regulation of protein metabolic process (GO:0051246)	2,02E-04
		circulatory system development (GO:0072359)	3,19E-04	regulation of biological process (GO:0050789)	2,02E-04
		regulation of molecular function (GO:0065009)	3,27E-04	regulation of angiogenesis (GO:0045765)	2,04E-04
		cellular response to oxygen-containing compound (GO:1901701)	3,59E-04	response to stimulus (GO:0050896)	2,24E-04
		positive regulation of inflammatory response (GO:0050729)	4,40E-04	positive regulation of multicellular organismal process (GO:0051240)	2,29E-04
		cellular response to stimulus (GO:0051716)	4,47E-04	negative regulation of protein metabolic process (GO:0051248)	2,32E-04
		regulation of cell proliferation (GO:0042127)	4,93E-04	vasculature development (GO:0001944)	2,35E-04
		regulation of cellular component movement (GO:0051270)	4,98E-04	blood vessel morphogenesis (GO:0048514)	2,38E-04
		regulation of developmental process (GO:0050793)	5,07E-04	regulation of cellular protein metabolic process (GO:0032268)	2,65E-04
		cell proliferation (GO:0008283)	5,44E-04	cellular response to organic substance (GO:0071310)	2,75E-04
		cell cycle process (GO:0022402)	5,57E-04	negative regulation of cellular protein metabolic process (GO:0032269)	2,80E-04
		biological_process (GO:0008150)	5,70E-04	cardiovascular system development (GO:0072358)	2,88E-04
		Unclassified (UNCLASSIFIED)	5,79E-04	tube development (GO:0035295)	3,16E-04
		positive regulation of biological process (GO:0048518)	6,36E-04	regulation of cellular process (GO:0050794)	4,36E-04
		regulation of cellular process (GO:0050794)	6,51E-04	regulation of cell motility (GO:2000145)	4,72E-04
		cell migration (GO:0016477)	6,76E-04	anatomical structure morphogenesis (GO:0009653)	5,01E-04
		organic substance biosynthetic process (GO:1901576)	7,14E-04	positive regulation of cell differentiation (GO:0045597)	5,17E-04
		regulation of biological process (GO:0050789)	7,41E-04	positive regulation of cell death (GO:0010942)	5,36E-04
		biosynthetic process (GO:0009058)	7,50E-04	regulation of cellular component organization (GO:0051128)	5,36E-04
		leukocyte activation (GO:0045321)	7,84E-04	regulation of molecular function (GO:0065009)	6,49E-04
		regulation of multicellular organismal process (GO:0051239)	7,87E-04	response to molecule of bacterial origin (GO:0002237)	7,00E-04
		cytokine-mediated signaling pathway (GO:0019221)	7,92E-04	regulation of cell death (GO:0010941)	7,02E-04
		positive regulation of defense response (GO:0031349)	8,94E-04	positive regulation of developmental process (GO:0051094)	7,63E-04
		response to oxygen-containing compound (GO:1901700)	9,29E-04	regulation of locomotion (GO:0040012)	7,91E-04
		cell motility (GO:0048870)	9,45E-04	regulation of apoptotic process (GO:0042981)	8,45E-04
		response to endogenous stimulus (GO:0009719)	9,52E-04	positive regulation of response to stimulus (GO:0048584)	8,54E-04
		localization of cell (GO:0051674)	9,58E-04	response to peptide (GO:1901652)	9,29E-04
		cellular developmental process (GO:0048869)	9,64E-04	cell death (GO:0008219)	9,60E-04
				regulation of localization (GO:0032879)	9,63E-04
