# Supplementary material for: An Early Neoplasia Index (ENI10), Based on Molecular Identity of CD10 Cells and Associated Stemness Biomarkers, is a Predictor of Patient Outcome in Many Cancers
Source: Cancer Res Commun. 2023 Sep 29;3(9):1966–80. doi: 10.1158/2767-9764.CRC-23-0196 (PMC10540743; doi:10.1158/2767-9764.CRC-23-0196)
Supplement: Supplementary Table S2 — shows the results of the CD10 IHC staining on a breast cancer tumor micro-array. [file crc-23-0196-s05.pdf]

Supplementary Table S2. Results of a CD10 immuno-histochemistry staining of a breast cancer TMA (Tumor Micro-Array).

	N	Luminal A<br>*N* =236	Luminal B<br>*N* =145	HER2 seul<br>*N* =12	Triple neg.<br>*N* =45	Combined<br>*N* =438	Test Statistic
CD10 (cat.)	383						$\chi^2_3 = 18.54, P < 0.001$
positive		1% (1)	1% (2)	9% (1)	11% (4)	2% (8)	
CD10 (% marked cells)	383						$\chi^2_{15} = 43.26, P < 0.001$
0		99% (196)	99% (135)	91% (10)	89% (34)	98% (375)	
20		1% (1)	0% (0)	0% (0)	0% (0)	0% (0)	
30		0% (0)	0% (0)	0% (0)	3% (1)	0% (0)	
70		0% (0)	0% (0)	0% (0)	5% (1)	1% (2)	
80		0% (0)	1% (1)	0% (0)	0% (0)	0% (0)	
90		0% (0)	1% (1)	1% (1)	3% (1)	1% (3)	
CD10 (stroma)	379						$\chi^2_3 = 1.78, P = 0.62$
positive		80% (158)	79% (106)	64% (7)	78% (28)	79% (299)	
