# Supplementary material for: An Early Neoplasia Index (ENI10), Based on Molecular Identity of CD10 Cells and Associated Stemness Biomarkers, is a Predictor of Patient Outcome in Many Cancers
Source: Cancer Res Commun. 2023 Sep 29;3(9):1966–80. doi: 10.1158/2767-9764.CRC-23-0196 (PMC10540743; doi:10.1158/2767-9764.CRC-23-0196)
Supplement: Supplementary Table S3 — shows the genes part of the ENI10, ENI10-GO and ENI10-SA molecular signatures. [file crc-23-0196-s06.pdf]

Supplementary Table S3. List of genes constituting the ENI10 signature and its subsets

ENI10

ADRB2	CCNG2	CYP1B1	FAM83D	IL1R2	NALCN	RAB2A	SMC4
AKR1B1	CD59	CYP4B1	FANCD2	IL1RL1	NAMPT	RANBP17	SNCAIP
AKR1B10	CDC25C	DAB2	FAP	KANK2	NCAPD3	RPL37A	SOD2
AKR1C1	CDCA3	DAPK1	FBXO5	KCNK5	NCAPG	RPS27L	SPAG5
ANLN	CDCA5	DCN	FIBIN	KIF14	NCAPG2	S100A4	SPC24
AOX1	CDK1	DDIAS	FLRT3	KIF18B	NCAPH	S100A7	SPC25
ASPM	CENPA	DDX17	FN1	KIF20A	NDC80	S100P	SRGN
ATAD2	CENPE	DEPDC1	FOXQ1	KIF23	NEK2	S1PR3	STIL
ATP6V0E1	CENPF	DEPDC1B	FPR1	KIF2C	NEURL1B	SAA2	SULT1E1
AURKA	CENPJ	DIAPH3	GALNT15	KIF4A	NNMT	SAMSN1	SUSD2
AURKB	CEP55	DKK1	GAS2L3	KPNA2	NUCKS1	SCNN1B	SUV39H1
BUB1	CFB	DLGAP5	GOLGA8A	LCN2	NUDCD2	SCNN1G	TACC3
BUB1B	CFLAR	EEF1D	GPR64	LIMCH1	NUSAP1	SERPINA5	TAF5
C1R	CKAP2	EHF	HEY1	MASTL	ORC6	SERPINE2	TMEM139
C1S	CKAP2L	ENO1	HJURP	MCM8	OSBPL7	SFTPB	TMEM194A
CASC5	CLIC3	ERAP1	HMGB2	METTL9	PAPSS2	SGOL2	TNFAIP6
CCDC71L	CNTN3	ESPL1	HMMR	MGLL	PDZK1IP1	SH3RF3	TOP2A
CCNA2	COL12A1	FAM64A	HPSE	MKI67	PI3	SHCBP1	TTK
CCNB1	COL8A1	FAM72A	HSP90B1	MLPH	PLSCR4	SLC39A8	UBE2C
CCNF	CXCL8	FAM76B	IFI44L	MME	PRC1	SLFN5	

ENI10-GO

ANLN	CCNA2	CENPF	FANCD2	KIF2C	NDC80	SPAG5	TTK
ASPM	CCNB1	CENPJ	FBXO5	KIF4A	NEK2	SPC24	UBE2C
AURKA	CDC25C	CEP55	GAS2L3	MASTL	NUSAP1	SPC25	
AURKB	CDCA5	CKAP2	KIF14	NCAPD3	ORC6	STIL	
BUB1	CDK1	DLGAP5	KIF18B	NCAPG	PRC1	SUSD2	
BUB1B	CENPA	ESPL1	KIF20A	NCAPG2	SGOL2	TACC3	
CASC5	CENPE	FAM83D	KIF23	NCAPH	SMC4	TOP2A	

ENI10-SA

BUB1	PRC1
BUB1B	SPAG5
CENPE	SPC24
CENPJ	SPC25
KIF23	STIL
KIF4A	TTK
NDC80	
