# Supplementary material for: An Early Neoplasia Index (ENI10), Based on Molecular Identity of CD10 Cells and Associated Stemness Biomarkers, is a Predictor of Patient Outcome in Many Cancers
Source: Cancer Res Commun. 2023 Sep 29;3(9):1966–80. doi: 10.1158/2767-9764.CRC-23-0196 (PMC10540743; doi:10.1158/2767-9764.CRC-23-0196)
Supplement: Supplementary Table S4 — shows the correlation between the ENI10 score and the IC50 of the indicated drugs in breast cancer cell lines from the "Genomics of Drug Sensitivity in Cancer Project". [file crc-23-0196-s07.pdf]

Supplementary Table S4. Correlation of ENI10 score with drug response of breast cancer Sanger cell lines.

CANCER	drug	n_cell_lines	r_IC50	pval_IC50	p_IC50_BH	signif.
BRCA	Bleomycin (10 uM)	45	-0.489586249305978	0.000641877912189661	0.120993986447751	yes
BRCA	IGFR_3801	37	-0.485114400178932	0.00234089832769402	0.142330134657947	suggestive
BRCA	Refametinib	86	-0.48225299578049	2.57973958996992E-06	0.000972561825418661	yes
BRCA	Cetuximab	44	-0.455565295933414	0.0018869419217173	0.142330134657947	suggestive
BRCA	Bleomycin (50 uM)	46	-0.444060602430349	0.0019920489484808	0.142330134657947	suggestive
BRCA	IC-87114	45	-0.413418561058339	0.00476142963352617	0.219926977719365	suggestive
