# Supplementary material for: An Early Neoplasia Index (ENI10), Based on Molecular Identity of CD10 Cells and Associated Stemness Biomarkers, is a Predictor of Patient Outcome in Many Cancers
Source: Cancer Res Commun. 2023 Sep 29;3(9):1966–80. doi: 10.1158/2767-9764.CRC-23-0196 (PMC10540743; doi:10.1158/2767-9764.CRC-23-0196)
Supplement: Supplementary Table S5 — shows the drugs corresponding to the targets indicated in Figures 2K and 5E of the main text. [file crc-23-0196-s08.pdf]

Supplementary Table S5. Correspondence between described targets and drugs

Target	Drug
LCK	A-770041
EGFR	Cetuximab
EphB4	EphB4_9721
IGF1-R	IGFR_3801
PARP1,2,5a	PARP_9482
PARP1,2,7	PARP_9495
MEK1/2	Refametinib
PARP5a, 5b	TANK_1366
LCK	WH-4-023
DSB inducer	Bleomycin
PI3K delta	IC-87114
