# Supplementary material for: An Early Neoplasia Index (ENI10), Based on Molecular Identity of CD10 Cells and Associated Stemness Biomarkers, is a Predictor of Patient Outcome in Many Cancers
Source: Cancer Res Commun. 2023 Sep 29;3(9):1966–80. doi: 10.1158/2767-9764.CRC-23-0196 (PMC10540743; doi:10.1158/2767-9764.CRC-23-0196)
Supplement: Supplementary Table S6 — shows the correlation between the ENI10 score and the IC50 of the indicated drugs in cancer cell lines from the "Genomics of Drug Sensitivity in Cancer Project". [file crc-23-0196-s09.pdf]

Supplementary Table S6. Correlation of ENI10 score with drug response of Sanger cell lines.

drug	n_cell_lines	r_IC50	pval_IC50	p_IC50_BH	signif.
PARP_9482	370	-0.216764322957314	0.0000260454997323774	0.000114860653819785	yes
EphB4_9721	667	-0.203645438740534	1.12522668869774E-07	1.12778402208114E-06	yes
A-770041	247	-0.192758015711124	0.00234506309628744	0.00642343369852647	suggestive
IGFR_3801	359	-0.187610836667159	0.000351424378517082	0.0011479863031558	yes
WH-4-023	239	-0.184644516636722	0.00418013371313056	0.01065571657509	suggestive
Cetuximab	687	-0.169870551255391	7.57375922086577E-06	0.0000407320465414854	yes
PARP_9495	669	-0.157238864033432	0.0000441111703256375	0.000181803982370151	yes
TANK_1366	733	-0.139411382655261	0.000152793986443239	0.000556183149525432	yes
Refametinib	1174	-0.0915526117920456	0.00168841256956994	0.00486660093581923	suggestive
